# Accelerated biological aging: unveiling the path to cardiometabolic multimorbidity, dementia, and mortality

**DOI:** 10.3389/fpubh.2024.1423016

**Published:** 2024-10-30

**Authors:** Yi He, Yu Jia, Yizhou Li, Zhi Wan, Yi Lei, Xiaoyang Liao, Qian Zhao, Dongze Li

**Affiliations:** ^1^Department of Neurology, Chengdu Seventh People’s Hospital, Chengdu, China; ^2^General Practice Ward/International Medical Center Ward, General Practice Medical Center, West China Hospital, Sichuan University, Chengdu, China; ^3^State Key Laboratory of Oral Diseases, National Clinical Research Center for Oral Diseases, National Center of Stomatology, West China School of Stomatology, Sichuan University, Chengdu, China; ^4^Department of Emergency Medicine, West China Hospital, Sichuan University, Chengdu, Sichuan, China

**Keywords:** biological aging, cardiometabolic multimorbidity, cardiometabolic diseases, mortality, dementia, disease trajectory

## Abstract

**Background:**

Cardiometabolic multimorbidity (CMM) and aging are increasing public health concerns. This prospective study used UK Biobank cohort to investigate the relationship between biological aging and the trajectory of CMM to dementia and mortality.

**Methods:**

CMM is the coexistence of at least two cardiometabolic diseases (CMD), including stroke, ischemic heart disease, and diabetes. Biological age was calculated using the KDM-BA and PhenoAge algorithms. Accelerated aging indicated biological age advances more rapidly than chronological age.

**Results:**

The study included 415,147 individuals with an average age of 56.5 years. During the average 11-year follow-up period, CMD-free individuals with accelerated aging had a significantly greater risk of CMD (KDM-BA, HR 1.456; PhenoAge, HR 1.404), CMM (KDM-BA, HR 1.952; PhenoAge, HR 1.738), dementia (KDM-BA, HR 1.243; PhenoAge, HR 1.212), and mortality (KDM-BA, HR 1.821; PhenoAge, HR 2.047) in fully-adjusted Cox regression models (*p* < 0.05 for all). Accelerated aging had adjusted HRs of 1.489 (KDM-BA) and 1.488 (PhenoAge) for CMM, 1.434 (KDM-BA) and 1.514 (PhenoAge) for dementia, and 1.943 (KDM-BA) and 2.239 (PhenoAge) for mortality in participants with CMD at baseline (*p* < 0.05 for all). CMM significantly mediated accelerated aging’s indirect effects on dementia by 13.7% (KDM-BA, HR) and 21.6% (PhenoAge); those on mortality were 4.7% (KDM-BA) and 5.2% (PhenoAge). The population attributable-risk of Life’s Essential 8 score (≥80 vs. <80) were 0.79 and 0.43 for KDM-BA and PhenoAge accelerated aging, respectively.

**Conclusion:**

Biological aging involves the entire trajectory of CMM from a CMD-free state to CMD, to CMM, and ultimately to dementia and death. Life’s Essential 8 may be a potential target to counter age acceleration.

## Introduction

Amidst the intensification of societal aging, cardiometabolic diseases (CMDs) have emerged as the most significant health challenges, with related deaths increasing by 56% from 1990 to 2019, reaching 15.7 million (1, 2). Cardiometabolic multimorbidity (CMM) refers to the coexistence of two or more CMDs, including conditions such as stroke, ischemic heart disease (IHD), and type 2 diabetes (T2D) (3). Populations with CMM have a two-fold increased mortality risk and a 12–15 year reduced life expectancy than those with single CMDs (1). The co-occurrence of multiple chronic CMDs has become increasingly common as the population ages (4, 5). Compared with people aged ≥40 years, the prevalence of CMM was 2.5-fold greater in people aged ≥60 years (6). Additionally, approximately one-third of older adults suffer from at least two co-morbid CMDs (7). Essentially, biological aging, which is associated with decreased metabolic rates, vascular stiffening, chronic inflammation, and oxidative stress, as well as the interplay of comorbidities, may underlie the progression of CMDs to CMM (8, 9). However, few studies have demonstrated the critical role of biological aging in this transition, and there is a notable lack of focus on biological aging as a mediator of health outcomes.

Aging is a complicated, gradual impairment of reserve function, recovery ability, and integrity of organs, tissues, and cells (10). The ideal measurement strategy for biological aging should include as many system indicators as possible (11). For predicting disease, the PhenoAge and Klemera–Doubal method Biological Age (KDM-BA) are the best-validated algorithms according to blood-chemistry–derived measures in multi-ethnic cohorts of older adults (12–15). Accelerated aging refers to the phenomenon where an individual’s biological age advances more rapidly than their chronological age. This concept goes beyond the mere passage of time, as it emphasizes the underlying biological processes and pathological changes. Thus, this study used both algorithms to test the association between biological aging and CMM.

Aging is the most influential factor in cognitive decline, regardless of dementia (16, 17). The 2020 Lancet Commission highlighted the need for a combination of cardiovascular and metabolic risk factors to minimize dementia and cognitive decline (18). CMM accelerates cognitive decline and increases the risk of cognitive impairment and dementia (19, 20). Thus, we hypothesize that accelerated biological aging is a pivotal factor in the transition from a CMD-free state to CMD, CMM, and ultimately to dementia and mortality.

Prolonging healthy aging improves the quality of life and reduces healthcare costs (10, 21, 22). High blood sodium levels, weight fluctuations, and a highly inflammatory diet are associated with the risk of accelerated aging (10, 23, 24). However, the contribution of cardiovascular health primordial prevention factors to biological aging is unclear (25). The American Heart Association’s Life’s Essential 8 (LE8) is a comprehensive metric that encapsulates eight key factors contributing to cardiovascular health, this framework include blood pressure, cholesterol, blood glucose, healthy diet, physical activity, weight, tobacco, and alcohol consumption (26). It serves as a critical tool for identifying risk factors and guiding interventions that can prevent or delay the onset of CMDs and mortality incident. Thus, we hypothesize that LE8 can serve as a preventive target to slow this aging process and mitigate adverse health outcomes.

Therefore, this study used data from the UK Biobank cohort to (1) investigate the relationship between accelerated aging and the risk of CMM, dementia, and mortality in healthy individuals; (2) analyze the relationship of accelerated aging with the risk of dementia and mortality in individuals with a single CMD or CMM; (3) clarify the mediating effect of CMM on accelerated aging leading to dementia or death; and (4) determine the relationship between the LE8 score and its components with accelerated aging.

## Methods

### Study design and population

The UK Biobank is an international health research cohort that included 502,655 UK adults (37–73 years old) between 2006 and 2010 (27). At baseline, the participants provided information on their lifestyle and health and their biological samples. For details of data collection.^1^ Data and materials can be obtained.^2^ All participants provided written informed consent before data collection. Ethics approval of UK Biobank study was approved by NHS National Research Ethics Service (16/NW/0274). The experimental protocols were established according to the ethical guidelines of the Helsinki Declaration. Written informed consent was obtained from individual or guardian participants. All methods were carried out in accordance with guidelines and regulations developed by UK Biobank. Data usage was approved by the Human Ethical Committee of the West China Hospital of Sichuan University (2023–1829).

This study included 415,147 participants after excluding those who withdrew from the UK Biobank study (*n* = 242), participants with dementia (*n* = 177) at baseline, participants without available data on biological age (*n* = 82,089), non-Caucasians (*n* = 3486), and individuals whose missing covariates were greater than 20% (*n* = 1756). The participants were divided into three groups according to their baseline status (Supplementary eFigure 1), as follows: Group 1: Participants without CMD (*n* = 373,400); Group 2: participants with at least one CMD (*n* = 41,747); and Group 3: all participants (*n* = 415,147).

### Assessment of CMDs, dementia, and all-cause mortality

Outcomes were ascertained using hospital records containing data on admissions and diagnoses obtained from the Hospital Episode Statistics for England, the Scottish Morbidity Record data for Scotland, and the Patient Episode Database for Wales. Diagnoses were recorded using the International Classification of Diseases–10th revision (ICD-10) coding system. The primary outcomes of this study were CMDs (stroke, IHD, or T2D), all-cause dementia (Alzheimer’s disease, vascular dementia, and other causes), and all-cause mortality. According to the ICD-10, we identified the outcomes: T2D (E11), IHD (I20–I25), stroke (I60–I69), and dementia (F00, F01, F02, F03, F05.1, G30, G31.1, and G31.8). Cases of all-cause death were identified by linking them to the national death registries. Follow-up time was calculated from the enrollment date to the earliest date of the first diagnosis of events (stroke, IHD, T2D, or dementia), death, or end of the current follow-up.

### Assessment of biological ages and age accelerations

Biological age was measured using the best-validated algorithms, KDM-BA and PhenoAge, which can be implemented with 12 blood chemistry traits, systolic blood pressure, and lung function data in the UK Biobank (12–14). Briefly, KDM-BA was computed from systolic blood pressure, forced expiratory volume in 1 s (FEV1), and seven other blood chemistry traits (albumin, alkaline phosphatase, creatinine, C-reactive protein, blood urea nitrogen, glycated hemoglobin, and total cholesterol). PhenoAge was computed from nine blood chemistries; four overlapped with KDM-BA (albumin, alkaline phosphatase, creatinine, C-reactive protein, glucose, mean cell volume, red cell distribution width, white blood cell count, and lymphocyte proportion). Two biological ages were constructed for different purposes. KDM-BA was computed from an algorithm derived from a series of regressions of nine individual biomarkers on chronological age in the reference population to quantify the decline in system integrity (28); PhenoAge was computed from an algorithm derived from multivariate analysis of mortality hazards to estimate the risk of death (29). Details of the selected traits and algorithms are provided in the Supplementary materials.

### Assessment of life’s essential 8 score

The LE8 score was calculated to assess cardiovascular health, including four biological (blood lipids, blood glucose, blood pressure, and body mass index) and four behavioral (smoking status, physical activity, diet, and sleep) targets (26). Detailed algorithms for calculating LE8 scores have been published and are provided in the Supplementary materials.

### Measurements of covariates

Age, sex, educational status, income, sleep duration, diet, drinking status, smoking status, physical activity, body mass index, systolic pressure, diastolic pressure, total cholesterol, high-density lipoprotein cholesterol, low-density lipoprotein cholesterol, triglycerides, and blood glucose were included as covariates to identify potential confounding factors.

### Statistical analysis

Parametric and non-parametric continuous variables are reported as medians (25th, 75th) or means ± standard deviations and were compared using analysis of variance or Mann–Whitney U test, respectively. Categorical variables are reported as n (%) and were compared using the chi-squared test. A total of 1.3% of covariates were missing, with income being the most significantly missing at 4.3%. Missing covariables were imputed with multivariate imputation using a chained equation (30). After 10 iterations, we generated 5 imputed datasets. Utilizing the ‘quickpred’ function from the ‘mice’ package in R, we identified predictors for each variable. We adapted the original function to include a maximum of 11 predictors, with the stipulation that sex must be included.

For Analysis 1, to investigate the impact of biological aging on the risk of outcome events over time, we used the Cox proportional hazards model to examine the prospective associations between baseline accelerated aging (PhenoAge and KDM-BA) and the incidence of CMDs, dementia, and all-cause mortality among participants without any CMDs at baseline. This model is designed to analyze time-to-event data, which is the time until an event of interest occurs, and is associated with a set of covariates. It is particularly useful for handling multiple covariates and can accommodate right-censored data, where the event of interest has not occurred by the end of the study period. In this study, the groups without accelerated aging were set as the reference group to calculate the hazard ratio (HR) and 95% confidence interval (CI) for the group with accelerated aging. Additionally, we calculated the increased relative risk associated with each additional year of aging acceleration. In these models, we adjusted for covariates, including age, sex, education status, income, sleep duration, diet, drinking status, smoking status, physical activity, body mass index, systolic pressure, diastolic pressure, total cholesterol, high-density lipoprotein cholesterol, low-density lipoprotein cholesterol, triglycerides, and blood glucose. Sensitivity analyses were conducted among individuals with >2 years of follow-up to avoid reverse causation and those without cardiovascular disease at baseline.

For Analysis 2, among participants with at least one CMD at baseline, Cox proportional hazard models were used to analyze the prospective associations between baseline biological aging and the incidence of dementia and all-cause mortality. Furthermore, the association between baseline biological aging and CMM incidence was examined in patients with a first CMD (T2D, IHD, or stroke). Similar subgroup and sensitivity analyses were performed. All Models were adjusted for these covariates.

For Analysis 3, based on the assumption that CMD or CMM is a crucial mediator between accelerated aging and dementia or death, we analyzed the degree to which CMD or CMM mediated this association using mediation analysis with structural equation modeling (31). Mediation Analysis is used to investigate the indirect effect of an independent variable (X) on a dependent variable (Y) through one or more mediator variables (M). It begins by analyzing the impact of X on M, then the impact of M on Y, and finally the total effect of X on Y. The mediating effect was calculated as the estimated indirect effect size divided by the estimated total effect size. The significance of the indirect effect is determined by constructing 95%CI or conducting hypothesis tests, and the results are shown as adjusted HRs and mediated proportions. For bootstrapping, 1000 iterations were performed to estimate the 95% bias-corrected confidence interval. The mediation percentage was calculated as log (estimated indirect effect)/log (estimated total effect).

For Analysis 4, to explore the potential factors that affect biological aging, we tested the cross-sectional associations of the LE8 score and each factor with the odds ratio (OR) of KDM-BA and PhenoAge biological aging at baseline using multivariate logistic regression and linear regression models. The relationships between the LE8 score and the KDM-BA and PhenoAge accelerations were analyzed in different age groups and sexes. All models were adjusted for sex, age, income, education, and eight LE8 factors. To visualize the changing trends in biological aging, chronological age, and LE8 score, 3D mesh plots were drawn using SigmaPlot 12.5 software with LOESS smoothing. Population attributable risk was calculated for LE8 and each individual component. All metrics were set as binary variables and simultaneously included in the model. When LE8 scores ≥80 or each component scores 100, these metrics were defined as the low-risk category.

All statistical analyses were performed using the R software 4.1.0 (Vienna, Austria). Statistical significance was set at *p* < 0.05 for all tests.

## Results

### Baseline characteristics and biological ages

The baseline characteristics of the groups are presented in Table 1. Among the 415,147 individuals (Group 3), the average age was 56.5 years (46.3% male). Group 1 included 373,400 individuals without baseline CMD; Group 2 comprised 41,747 individuals with ≥CMD. Group 2 had a greater value or likelihood of cardiometabolic risk factors, including low physical activity, unhealthy sleep, smoking, high blood pressure, high lipids, and high glucose levels than Group 1.

**Table 1:** Population baseline characteristics in UK Biobank study.

Characteristic	Group 1^a^	Group 2^a^	Group 3^a^
	(*N* = 373,400)	(*N* = 41,747)	(*N* = 415,147)
Demographic and behavior variables			
Age, years	56.05 (8.10)	60.59 (6.78)	56.50 (8.09)
Male sex, n (%)	165037 (44.2%)	27037 (64.8%)	192074 (46.3%)
Education, n (%)			
College or University degree	125771 (33.7%)	9435 (22.6%)	135206 (32.6%)
A levels/AS levels or equivalent	42639 (11.4%)	3708 (8.9%)	46347 (11.2%)
O levels/GCSEs or equivalent	101926 (27.3%)	9894 (23.7%)	111820 (26.9%)
Other (e.g. NVO, nursing, missing)	103064 (27.6%)	18710 (44.8%)	121774 (29.3%)
Income, US, n (%)			
<£18000	88053 (23.8%)	16936 (40.6%)	105989 (25.5%)
£18000 to £52000	188672 (50.5%)	19343 (46.3%)	208015 (50.1%)
>£52000	95675 (25.6%)	5468 (13.1%)	101143 (24.4%)
MET, hours/week	33.00 (14.25–65.90)	11.30 (29.33–62.50)	32.55 (13.90–65.54)
Sleep 7–8 h/d, n (%)	255024 (68.3%)	24714 (59.2%)	279738 (67.4%)
Healthy diet^b^, n (%)	90637 (24.3%)	13150 (31.5%)	103787 (25.0%)
Drinking, n (%)			
Never	15380 (4.1%)	2666 (6.4%)	18046 (4.3%)
Former	12213 (3.3%)	2575 (6.2%)	14788 (3.6%)
Current	345807 (92.6%)	36506 (87.4%)	382313 (92.1%)
Smoking, n (%)			
Never	208919 (56.0%)	17985 (43.1%)	226904 (54.7%)
Former	125884 (33.7%)	18790 (45.0%)	144674 (34.8%)
Current	38597 (10.3%)	4972 (11.9%)	43569 (10.5%)
Physiological and lab variables			
Body mass index, kg/m^2^	27.1 ± 4.61	29.65 ± 5.27	27.40 ± 4.74
Diastolic blood pressure, mmHg	82.39 ± 10.14	80.76 ± 10.07	82.23 ± 10.14
HDL-C, mmol/l	1.47 ± 0.38	1.25 ± 0.34	1.45 ± 0.38
LDL-C, mmol/l	3.64 ± 0.83	2.79 ± 0.80	3.56 ± 0.87
Triglycerides, mmol/l	1.72 ± 1.01	1.96 ± 1.14	1.74 ± 1.02
Components of biological ages			
FEV1^c^, L	2.84 ± 0.80	2.81 ± 0.80	2.82 ± 0.80
Systolic blood pressure^c^, mmHg	137.56 ± 18.66	139.84 ± 18.14	137.79 ± 18.62
Total cholesterol^c^, mmol/l	5.81 ± 1.09	4.63 ± 1.07	5.69 ± 1.14
Glycated hemoglobin^c^, %	35.11 ± 4.57	36.13 ± 6.81	36.08 ± 6.69
Blood urea nitrogen^c^, mmol/l	5.35 ± 1.31	5.40 ± 1.39	5.40 ± 1.39
Lymphocyte^d^, %	29.05 ± 7.43	28.94 ± 7.45	28.92 ± 7.48
Mean cell volume^d^, fL	82.90 ± 5.29	82.89 ± 5.25	82.85 ± 5.33
Blood glucose^d^, mmol/l	4.99 ± 0.81	6.34 ± 2.74	5.12 ± 1.23
Red cell distribution width^d^, %	13.47 ± 0.96	13.50 ± 0.99	13.49 ± 0.98
White blood cell count^d^, cells/mL	6.81 ± 1.90	6.88 ± 1.98	6.88 ± 1.94
Albumin^cd^, g/dL	45.30 ± 2.60	45.17 ± 2.62	45.23 ± 2.62
Creatinine^cd^, mmol/l	71.75 ± 15.25	72.18 ± 16.76	72.35 ± 16.43
C-reactive protein^cd^, mmol/l	1.29 (0.64–2.68)	1.34 (0.66–2.80)	1.32 (0.65–2.75)
Alkaline phosphatase^cd^, U/L	83.00 ± 25.74	83.60 ± 25.76	83.51 ± 26.20
Biological ages			
KDM-BA biological age, years	48.94 ± 10.07	54.91 ± 9.64	49.54 ± 10.19
KDM-BA accelerated aging^e^, n (%)	42381 (11.4%)	8626 (20.7%)	51007 (12.3%)
PhenoAge biological age, years	50.64 ± 9.53	58.64 ± 9.41	51.44 ± 9.81
PhenoAge accelerated aging^e^, n (%)	44074 (11.8%)	13033 (31.2%)	57107 (13.8%)

Values are expressed as n (%), mean (SD), and median (25–75th). MET, metabolic equivalent task; HDL-C, high density lipoprotein cholesterol; LDL-C, low density lipoprotein cholesterol; FEV1, forced expiratory volume in the first second.

a Group 1: population without cardiometabolic disease at baseline. Group 2: population with at least one cardiometabolic disease at baseline. Group 3: Total population of Group 1 plus Group 2.

b Healthy diet defined as highest quartile of Dietary Approaches to Stop Hypertension diet score.

c Employed to construct KDM-BA.

d Employed to construct PhenoAge.

e Accelerated aging defined as biological age higher than chronological age.

At baseline, Group 1 had a lower biological age (48.9–50.6 vs. 54.9–58.6 years) and ratio of accelerated aging (11.4–11.8% vs. 20.7–31.2%) than Group 2, regardless of the KDM-BA or PhenoAge algorithms (Table 1). Chronological age correlated highly with biological age (Pearson coefficient: 0.75 for KDM-BA and 0.85 for PhenoAge) in all participants. Biological ages and age acceleration calculated by KDM-BA and PhenoAge had significant correlations (Pearson coefficient: 0.77 and 0.38, respectively).

### Analysis 1: Association of biological aging with CMD, CMM, dementia, and mortality in CMD-free individuals

**Figure 1:**
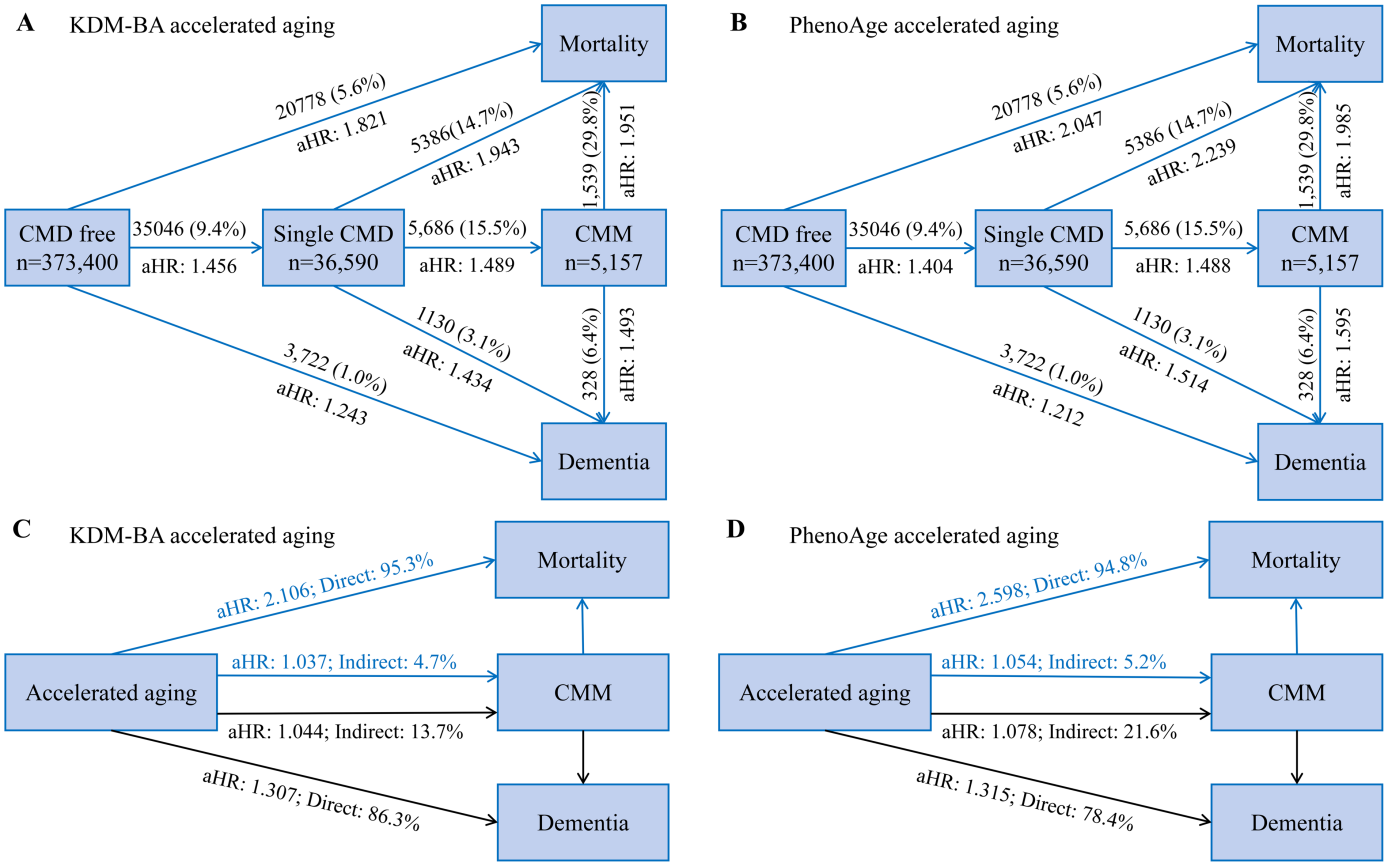
The panoramic trajectory of accelerated aging: from CMD to CMM, eventually developing into dementia and death. (A,B) Summary and comparison of the risks of dementia and death for individuals without CMD (Analysis 1), with CMD (Analysis 2), and with CMM (Analysis 3) are presented, respectively. Cox proportional hazards models were conducted to examine the prospective associations. (C,D) Mediation effect of CMM on relationship of accelerated aging with dementia and mortality. Indirect effect indicates the mediating effect of CMM on association between accelerated aging, dementia and mortality. Direct effect indicates total effect subtracted indirect effect. Model adjustment is the same as Table 2. The percentage was calculated by log (estimated indirect effect)/log (estimated total effect). CI, confidence interval; CMD, cardiometabolic disease; CMM, cardiometabolic multimorbidity; aHR, adjusted hazards ratio.

During an average of 11.7 years of follow-up, 35046 (9.4%), 3914 (1.0%), and 3722 (1.0%) CMD-free individuals developed CMD, CMM, and dementia, respectively, while 20778 (5.6%) died. In the fully adjusted Cox proportional hazards model (Table 2; Figure 1), CMD-free individuals with accelerated aging had greater risk of any CMD (KDM-BA, HR 1.456, 95%CI 1.414–1.500; PhenoAge, HR 1.404, 95%CI 1.366–1.444), CMM (KDM-BA, HR 1.952, 95%CI 1.802–2.114; PhenoAge, HR 1.738, 95%CI 1.612–1.874), all-cause dementia (KDM-BA, HR 1.243, 95%CI 1.121–1.378; PhenoAge, HR 1.212, 95%CI 1.106–1.329), and all-cause mortality (KDM-BA, HR 1.821, 95%CI 1.753–1.892; PhenoAge, HR 2.047, 95%CI 1.980–2.116). This study suggests that, in an ideal scenario, reversing the aging process could potentially prevent 31% of CMD, 49% of CMM, 19% of dementia, and 51% of mortality events. Moreover, accelerated aging was associated significantly with diabetes, ischemic heart disease, and stroke (*p* < 0.001). These associations were weaker in the older adults and males (P for interaction <0.05; Supplementary eTables 4–7). In the sensitivity analyses, participants with <2 years of follow-up or any cardiovascular disease were excluded, and the association between biological aging and these adverse events was consistent (*p* < 0.001; Supplementary eTables 8–9). Similar analyses and results for age acceleration were presented.

**Table 2:** Accelerated aging was significantly associated with CMD, CMM, dementia, and mortality among individuals who were free of CMD at baseline.

Biological age	KDM-BA			PhenoAge		
	Case/number (%)	aHR (95%CI)	*p*	Case/number (%)	aHR (95%CI)	*p*
Diabetes	13459/373400 (3.6%)	–	–	13459/373400 (3.6%)	–	–
Non-accelerated aging	9409/331019 (2.8%)	Reference	–	9700/329326 (2.9%)	Reference	–
Accelerated aging	4050/42381 (9.6%)	1.986 (1.902–2.074)	<0.001	3759/44074 (8.5%)	1.629 (1.563–1.698)	<0.001
Aging acceleration (per 1 year)	–	1.047 (1.044–1.050)	<0.001	–	1.040 (1.037–1.043)	<0.001
Ischemic heart disease	23976/373400 (6.4%)	–	–	23976/373400 (6.4%)	–	–
Non-accelerated aging	19668/331019 (5.9%)	Reference	–	19682/329326 (6.0%)	Reference	–
Accelerated aging	4308/42381 (10.2%)	1.296 (1.249–1.344)	<0.001	4294/44074 (9.7%)	1.329 (1.284–1.376)	<0.001
Aging acceleration (per 1 year)	–	1.020 (1.018–1.023)	<0.001	–	1.028 (1.026–1.031)	<0.001
Stroke	5588/373400 (1.6%)	–	–	5588/373400 (1.6%)	–	–
Non-accelerated aging	4589/331019 (1.4%)	Reference	–	4534/329326 (1.4%)	Reference	–
Accelerated aging	999/42381 (2.4%)	1.379 (1.278–1.489)	<0.001	1054/44074 (2.4%)	1.508 (1.405–1.617)	<0.001
Aging acceleration (per 1 year)	–	1.024 (1.019–1.028)	<0.001	–	1.036 (1.031–1.041)	<0.001
Single cardiometabolic disease	35046/373400 (9.4%)	–	–	35046/373400 (9.4%)	–	–
Non-accelerated aging	28014/331019 (8.5%)	Reference	–	28185/329326 (8.6%)	Reference	–
Accelerated aging	7032/42381 (16.6%)	1.456 (1.414–1.500)	<0.001	6861/44074 (15.6%)	1.404 (1.366–1.444)	<0.001
Aging acceleration (per 1 year)	–	1.027 (1.025–1.029)	<0.001	–	1.032 (1.030–1.034)	<0.001
Cardiometabolic multimorbidity	3914/373400 (1.0%)	–	–	3914/373400 (1.0%)	–	–
Non-accelerated aging	2778/331019 (0.8%)	Reference	–	2816/329326 (0.9%)	Reference	–
Accelerated aging	1136/42381 (2.7%)	1.952 (1.802–2.114)	<0.001	1098/44074 (2.5%)	1.738 (1.612–1.874)	<0.001
Aging acceleration (per 1 year)	–	1.047 (1.042–1.052)	<0.001	–	1.048 (1.042–1.053)	<0.001
All-cause dementia	3722/373400 (1.0%)	–	–	3722/373400 (1.0%)	–	–
Non-accelerated aging	3224/331019 (1.0%)	Reference	–	3135/329326 (1.0%)	Reference	–
Accelerated aging	498/42381 (1.2%)	1.243 (1.121–1.378)	<0.001	587/44074 (1.3%)	1.212 (1.106–1.329)	<0.001
Aging acceleration (per 1 year)	–	1.016 (1.010–1.021)	<0.001	–	1.015 (1.008–1.021)	<0.001
All-cause mortality	20778/373400 (5.6%)	–	–	20778/373400 (5.6%)	–	–
Non-accelerated aging	16634/331019 (5.0%)	Reference	–	15542/329326 (4.7%)	Reference	–
Accelerated aging	4144/42381 (9.8%)	1.821 (1.753–1.892)	<0.001	5236/44074 (11.9%)	2.047 (1.980–2.116)	<0.001
Aging acceleration (per 1 year)	–	1.044 (1.041–1.046)	<0.001	–	1.063 (1.061–1.065)	<0.001

Accelerated aging indicates that biological age was greater than chronological age. Aging acceleration indicates biological age minus chronological age. Cox regression models were adjusted by age, sex, education, income, body mass index, physical activity, sleep duration, healthy diet, smoking status, drinking status, diastolic blood pressure, systolic blood pressure, high density lipoprotein cholesterol, low density lipoprotein cholesterol, triglycerides, total cholesterol, and blood glucose. CMD, cardiometabolic disease; CMM, cardiometabolic multimorbidity; aHR, adjusted hazard ratio.

### Analysis 2: Association of biological aging with CMM, dementia, and mortality in CMD individuals

**Figure 2:**
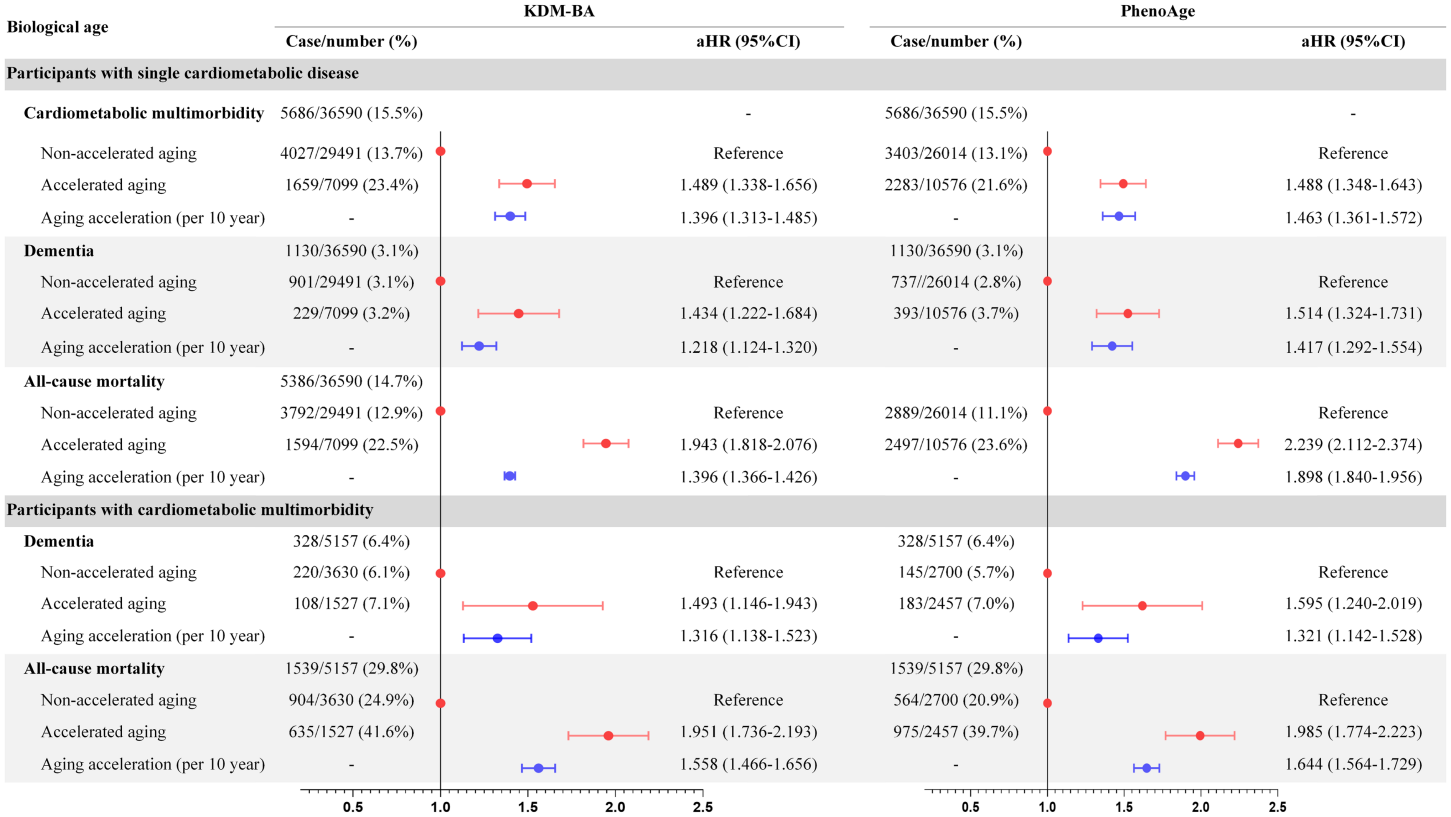
Association of biological aging with CMM, dementia, and mortality in CMD individuals. CMD, cardiometabolic disease; CMM, cardiometabolic multimorbidity.

During the average of 11.2 years follow-up, 5686 (15.5%) and 1130 (3.1%) cases of single baseline CMD developed CMM and dementia, respectively, while 5386 (14.7%) died; in those individuals, accelerated aging had adjusted HR of 1.489 (KDM-BA, 95%CI 1.338–1.656) and 1.488 (PhenoAge, 95%CI 1.348–1.643) for CMM, adjusted HRs of 1.493 (KDM-BA, 95%CI 1.146–1.943) and 1.195 (PhenoAge, 95%CI 0.940–1.519) for all-cause dementia, and adjusted HRs of 1.951 (KDM-BA, 95%CI 1.736–2.193) and 1.985 (PhenoAge, 95%CI 1.774–2.223) for all-cause mortality, respectively (Figures 1, 2). In individuals with baseline CMM, accelerated aging was independently associated with dementia (HR: 1.493 and 1.595, respectively; *p* < 0.001) and mortality (HR: 1.951 and 1.985, respectively; *p* < 0.001). These results underscore that in individuals with CMD, the detrimental effects of accelerated aging may be magnified, significantly increasing their risk of dementia by approximately half and doubling their risk of mortality. These associations were weaker in the older adults or males (*p* < 0.001; Supplementary eTables 11–13) and participants with follow-up of >2 years (Supplementary eTable 14). Accelerated aging was associated significantly with CMM, all-cause dementia, and all-cause mortality in individuals with DM, IHD, or stroke (Supplementary eTable 15).

### Analysis 3: Effect of CMM on the relationship of biological aging with dementia and mortality

In structural equation modeling (Supplementary eTables 16–20), the indirect effects of accelerated aging on all-cause dementia mediated by CMM were 13.7% (KDM-BA, aHR 1.044) and 21.6% (PhenoAge, aHR 1.078); the indirect effects on all-cause mortality were 4.7% (KDM-BA, aHR 1.037) and 5.2% (PhenoAge, aHR 1.054). Significant mediating effects of CMD on biological aging were observed for dementia (intermediate value 22.9–37.9%, *p* < 0.001) and mortality (intermediate value 7.8–9.4%, *p* < 0.001; Figure 2). This statistical value suggests that one-third of the dementia caused by accelerated aging can be explained by CMDs. However, while CMDs are also a significant mediator in the relationship between accelerated aging and mortality, the mediation effect size is notably lower, approximately 5%, compared to dementia. Diabetes mellitus, IHD, and stroke also significantly mediated dementia and mortality. To emphasize the risks of dementia and death for individuals without CMD, with CMD, and with CMM, respectively, a comprehensive presentation and comparison were shown in Figure 1. According to the results, the HRs for dementia and death were higher in individuals with CMD or CMM compared to those without CMD. This indicated that accelerated aging had a stronger association with health outcomes in the context of CMD.

### Analysis 4: Relationship between the life essential 8 score and biological aging

**Figure 3:**
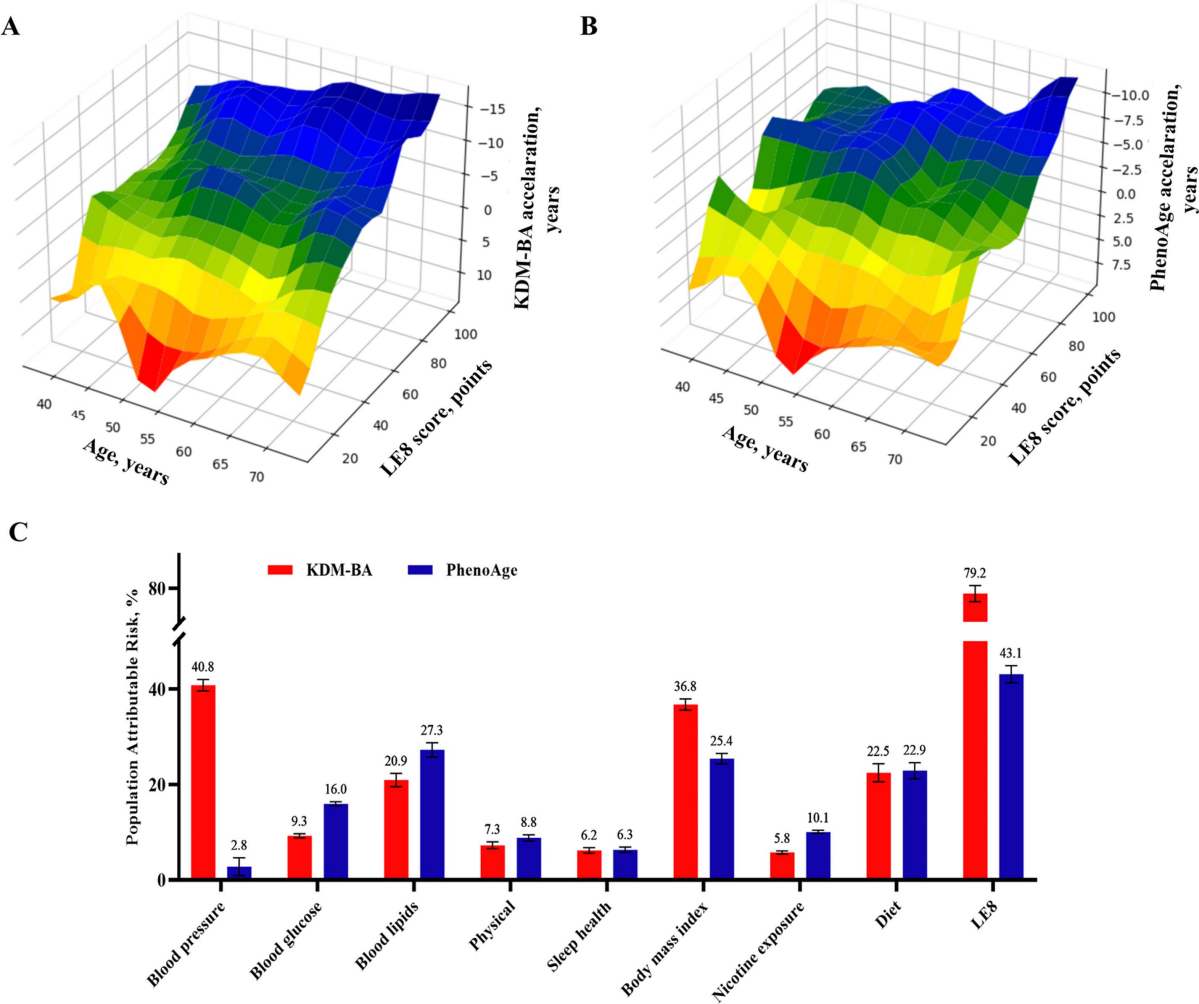
Dose–response relationship and crucial contribution of LE8 to biological aging. (A,B) 3D Mesh Plots visualizing differences between accelerated age, chronological age, and LE8 score. (C) Populations Attributable risk of LE8 score (≥80 vs. <80) and each risk factor for accelerated aging. LE8, life’s essential 8.

Individuals with low LE8 score (0–49) had adjusted OR of 16.615 (95%CI: 15.589–17.709) and 4.190 (95%CI: 4.021–4.365) for accelerated aging as calculated by KDM-BA and PhenoAge (Supplementary eTable 21), respectively, compared to those with a high score (80–100). These associations were weaker in the older adults or males (*p* < 0.001; Supplementary eTable 22). Moreover, 3D mesh graph visualization showed that participants with lower LE8 scores or chronological ages had greater aging acceleration and a greater likelihood of accelerated aging (Figure 3; Supplementary eFigure 3). Each LE8 component was an independent factor of biological aging (Supplementary eTable 23). The LE8 population attributable risk score was 0.79 (95%CI: 0.78–0.80) and 0.43 (95%CI: 0.41–0.45) for KDM-BA and PhenoAge accelerated aging, respectively; among the LE8 components, blood pressure and blood lipids were the main contribution factors to accelerated aging algorithm (Figure 3). This implies that among numerous aging risk factors, LE8 carries a very high weight. The data can still suggest that if the cardiovascular health indicated by LE8 is protected, it may be possible to prevent aging in 80% of the population at risk.

## Discussion

Using data from a community-based large-sample cohort from the UK Biobank, we found accelerated aging elevated risk of CMD by 40–45%, risk of CMM by 74–95%, risk of dementia by 21–24%, and risk of mortality by 82–105% in individuals without any CMD. While, for individuals with CMD, accelerated aging had about 50 and 100% higher risk of dementia and mortality, respectively. Regardless of baseline CMD status, individuals with biological aging had a significantly increased risk of all-cause dementia and mortality, partly (5–38%) mediated by CMD or CMM. These results suggest that aging plays a substantial role in the occurrence, progression, and prognosis of CMM, and that inhibiting the acceleration of aging may improve the adverse outcomes of dementia and death in one-third and one-half of the population, respectively. Considering biological aging is a more valuable intervention target than treating any single disease, we found the cardiovascular health assessment tool LE8 recommended by the AHA has a 41–80% population-attributed risk for biological aging, indicating cardiovascular health primordial prevention is an important strategy for suppressing biological aging.

Age acceleration, the epigenetic clock measured using whole blood DNA methylation, and shortened leukocyte telomere length are CMD risk factors and related positively to increased CVD (32, 33). Epigenetic age acceleration is a robust marker of myocardial infarction and cardiovascular disease mortality risk (34). Leukocyte telomere length negatively correlates with an increased risk of myocardial infarction and cardiovascular death (35). This study expands on the discovery that biological age acceleration measured using blood biochemical parameters is associated with CMD (including diabetes, stroke, and IHD) and CCM.

Diabetes mellitus was associated with a 37% increase in the leukocyte telomere-length attrition risk (35). Disrupted regulation of sirtuins and the mammalian target of rapamycin, which is related to longevity, induces abnormal glucose metabolism (36, 37). Therefore, a bidirectional relationship may exist between diabetes and biological aging. We found aging promotes CMD development toward CCM, especially stroke, and the transition to comorbidities.

Aging decreases life expectancy; biological age is a stronger predictor of age-related adverse events than chronological age, making it a valuable tool in precision medicine (29). Consistent with previous results, the acceleration of phenotypic age was associated with all-cause mortality in patients with diabetes. We also found individuals with other CMDs show a significant association between biological aging and mortality. Age is an important factor for cognitive decline; brain aging is an important initiating factor for dementia (38–40). However, no strong evidence exists regarding accelerated epigenetic aging associated with dementia or mild cognitive impairment (41). Herein, phenotypic age acceleration was a robust predictive factor for dementia independent of chronological age and traditional risk factors in participants with different CMD statuses.

The effects of CMD and CMM on dementia and mortality have been validated, but studies fail to assess the effect of biological aging on different CMM transition stages, including the transformation from CMD-free to CMD, then CMM, dementia, and death. We applied a marginal structural approach to test the indirect effects of accelerated aging on all-cause dementia and CMM-mediated mortality. We suggest that CMM is a critical link in age-related adverse events.

Several factors explain the relationship between biological aging and CMM. Cardiometabolic disorders, including arterial hypertension, dyslipidemia, insulin resistance, and hyperglycemia, share common pathophysiological mechanisms with aging (4, 42). For example, insulin resistance is mechanically induced by inflammation, oxidative stress, immune disorders, which are also complex biological processes involved in aging (43–45). Additionally, the blood vessels themselves undergo age-related changes, such as stiffening of the arteries, which can increase the risk of hypertension and cardiovascular diseases (46). Certain genetic predispositions and epigenetic modifications that accumulate over time can influence the rate of biological aging and the susceptibility to CMM (47). Moreover, Aging manifests as an impairment of the reserve and recovery ability of organs, tissues, and cells; thus, aging individuals are more susceptible to adverse cardiometabolic substrates, leading to progression from sustained chronic injury to CMD and comorbidities (42, 48). Demonstrating these potential mechanisms may promote the clinical application of biological aging concepts and contribute to precise individual health assessments, identifying high-risk populations before disease onset and implementing early intervention strategies.

Studies have stressed the protective effects of behavioral and environmental factors on vascular aging, including PM2.5, caloric restriction and fasting, physical exercise, smoking cessation, healthy diet, and low sodium intake (49–52). The American Heart Association included eight modifiable risk factors to optimize cardiovascular health when updating the LE8 (26). However, the significance of LE8 adherence for preventing biological aging remains unclear. Our results showed that 8 factors were independently associated with accelerated aging. Blood pressure, blood lipid levels, BMI, and diet were the main accelerated aging factors; LE8 adherence may counter aging. These results emphasize the importance of new perspectives for cardiovascular health prevention. Given this, future studies should design randomized controlled trials to test comprehensive intervention measures targeting the LE8 score. And during the follow-up process, continuous measurement of phenotypic age, telomere length, CMD events and death should be employed to verify the effectiveness and effect size of cardiovascular health in inhibiting the aging process. Additionally, preclinical experiments are equally important and may provide novel insights into how to inhibit the increase of phenotypic age at the molecular and cellular levels, as well as counteract the onset and progression of CMD caused by aging.

The study limitations include, first, the lack of repeated measurements of biological age, possibly explaining the significance of biological aging in the CMM disease trajectory. This defect is also limited to cross-sectional rather than prospective analysis of biological age’s influencing factors. Second, although the UK Biobank cohort enrolled the largest possible population, the response rate was only 5%; most were Caucasians, and the health status of these individuals was better than that of the general population (53). The CMM incidence rate was significantly lower than that in the Chinese cohort (0.9% vs. 6.3%) (5). Therefore, the universality of the results must be verified. Third, although potential confounding factors were corrected as much as possible, residual confounding factors may have affected the results. For example, the presence of other chronic diseases may affect the speed of aging and the occurrence of age-related diseases. The interaction between genes and environmental factors may affect aging, but this interaction is often complex and difficult to measure (47). Fourth, our models were adjusted for numerous cardiometabolic risk factors; biological age may have been affected by subclinical CVD at baseline; therefore, we cannot exclude the possibility of reverse causation. Fifth, although adjustments were made for many cardiometabolic risk factors, baseline biological age may be affected by subclinical CVD. In addition, diabetes promotes aging, meaning reverse causality is possible. Sixth, this study did not use epigenetic DNA methylation or telomere length to assess biological aging. Finally, considering that we conducted multiple tests in the main analysis (two measurements of biological age and multiple outcomes), our results should be interpreted cautiously and replicated in other study populations.

## Conclusion

Biological aging measured by the blood biochemical index was a strong risk marker for CMD, CMM, dementia, and death, independent of chronological age and other traditional cardiovascular risk factors. Notably, biological aging involved whole trajectory of CMM from a disease-free state to single CMD, and subsequently to CMM, and ultimately to dementia and death. Given the robust correlation and significant impact of LE8 on biological aging, this study offers avenues for future research, including the design of comprehensive interventions targeting LE8 in clinical trials to verify the effects of cardiovascular health interventions on inhibiting aging, CMD, and related adverse events. This also encompasses preclinical experiments aimed at developing new anti-aging targeted drugs from the perspective of cardiovascular health, to suppress the critical trajectory of biological aging that leads to CMM, dementia, and mortality.

## Data Availability

The original contributions presented in the study are included in the article/Supplementary material, further inquiries can be directed to the corresponding authors.

## Footnotes

^1^ http://www.ukBiobank.ac.uk

^2^ https://ukBiobank.dnanexus.com/panx/projects
