# Supplementary material for: Accelerated biological aging: unveiling the path to cardiometabolic multimorbidity, dementia, and mortality
Source: Front Public Health. 2024 Oct 30;12:1423016. doi: 10.3389/fpubh.2024.1423016 (PMC11559589; doi:10.3389/fpubh.2024.1423016)
Supplement: Supplementary file 1 [file Supplementary_file_1.docx]

**Supplementary Materials**

**Accelerated biological aging, related to life essential 8, leads to the trajectory of cardiometabolic multimorbidity to dementia and mortality**

**Table of Contents**

[Supplementary Method 3](#_Toc26608)

[*History of cardiovascular disease, diabetes, and dementia* 3](#_Toc30195)

[*Assessment of biological ages and age accelerations* 3](#_Toc13925)

[*Assessment of Life’s Essential 8 score* 5](#_Toc5585)

[Reference 6](#_Toc13497)

[Supplementary Figure and Table Legends 7](#_Toc554)

[eFigure 1. Study flow chart. CMD, cardiometabolic disease; CMM, cardiometabolic multimorbidity. 9](#_Toc13896)

[eFigure 2. Correlation heat map of chronological age, biological ages, and age accelerations. 10](#_Toc13257)

[eFigure 3. Relationship between LE8 score and biological aging at different ages. 11](#_Toc24616)

[eTable 1. Definition of the DASH diet score in the UK Biobank. 12](#_Toc21780)

[eTable 2. Methods for evaluating each individual Life's Essential 8 score in the UK Biobank. 13](#_Toc3623)

[eTable 3. Full names and data field IDs of variables for the construction of biological ages. 15](#_Toc18844)

[eTable 4. Subgroup analysis for the association of KDM-BA biological aging with CMD, CMM, dementia, and mortality in individuals with different chronological ages. 16](#_Toc6306)

[eTable 5. Subgroup analysis for the association of PhenoAge biological aging with CMD, CMM, dementia, and mortality in individuals with different chronological ages. 18](#_Toc18196)

[eTable 6. Subgroup analysis for the association of KDM-BA biological aging with CMD, CMM, dementia, and mortality in males. 20](#_Toc18978)

[eTable 7. Subgroup analysis for the association of PhenoAge biological aging with CMD, CMM, dementia, and mortality in females. 22](#_Toc13047)

[eTable 8. Sensitivity analysis for the association of biological aging with CMD, CMM, dementia, and mortality in CMD-free individuals after excluding individuals who had been followed up for less than 2 years. 24](#_Toc2796)

[eTable 9. Sensitivity analysis for the association of biological aging with CMD, CMM, dementia, and mortality in CMD and CVD free individuals. 26](#_Toc21094)

[eTable 10. Subgroup analysis for the association of KDM-BA biological aging with CMM, dementia, and mortality in CMD individuals with different ages. 28](#_Toc14808)

[eTable 11. Subgroup analysis for the association of PhenoAge biological aging with CMM, dementia, and mortality in CMD individuals with different ages. 30](#_Toc1551)

[eTable 12. Subgroup analysis for the association of KDM-BA biological aging with CMM, dementia, and mortality in CMD individuals with different genders. 32](#_Toc9525)

[eTable 13. Subgroup analysis for the association of PhenoAge biological aging with CMM, dementia, and mortality in CMD individuals with different genders. 34](#_Toc23056)

[eTable 14. Sensitivity analysis for the association of PhenoAge biological aging with CMM, dementia, and mortality in CMD individuals after excluding individuals who had been followed up for less than 2 years. 36](#_Toc27527)

[eTable 15. Association of biological aging with CMM, dementia, and mortality in individuals with diabetes, stroke, or ischemic heart disease. 38](#_Toc12856)

[eTable 16. Mediation impact of CMM on relationship of accelerated biological aging with dementia, and mortality. 40](#_Toc2903)

[eTable 17. Mediation impact of CMD on relationship of accelerated biological aging with dementia, and mortality. 41](#_Toc23830)

[eTable 18. Mediation impact of diabetes on relationship of accelerated biological aging with dementia, and mortality. 42](#_Toc25204)

[eTable 19. Mediation impact of ischemic heart disease on relationship of accelerated biological aging with dementia, and mortality. 43](#_Toc27828)

[eTable 20. Mediation impact of stroke on relationship of accelerated biological aging with dementia, and mortality. 44](#_Toc8282)

[eTable 21. Relatinship between LE8 score and biological aging according to multivariate Logistic regression. 45](#_Toc27118)

[eTable 22. Subgroup analysis for relationship between LE8 score and biological aging according to age and gender. 46](#_Toc3214)

[eTable 23. Relatinship between LE8 score and biological aging according to multivariate linear regression. 47](#_Toc24520)

# Supplementary Method

# *History of cardiovascular disease, diabetes, and dementia*

Cardiovascular disease outcomes were collected via medical history and linkage to data on hospital admissions, questionnaires, and death register data. IHD is defined by ICD-10 code I21-I25; stroke is defined by ICD-10 code I60-I64; heart failure is defined by ICD-10 code I110, I113 and I50.

Diabetes includes Type 1 and Type 2 diabetes, which are defined as self-reported disease history and the ICD-10 codes (E10).

All-cause dementia includes Alzheimer’s disease, vascular dementia and other causes, which are defined as self-reported disease history and the ICD-10 codes (F00, F01, F02, F03, F05.1, G30, G31.1, and G31.8).

***Assessment of biological ages and age accelerations***

The individual's KDM-BA prediction corresponds to their age at which their physiological functions are generally normal. KDM-BA is derived from a series of regressions of individual biomarkers to age in the reference population ^[1]^. This equation obtains information from n time age regression lines regressed on n biomarkers. The calculation formula is:

$$KDM-BA_{EC}=\frac{\sum_{i=1}^{n} \left( x_{i}-q_{i} \right)\frac{k_{i}}{s_{i}^{2}}+\frac{CA}{S_{BA}^{2}}}{\sum_{i=1}^{n} \left( \frac{k_{i}}{s_{i}} \right)^{2}+\frac{1}{S_{BA}^{2}}}$$

X is the value of the biomarker measured for an individual. The estimation of k, q, and s for each biomarker parameter is based on the regression of biomarkers with real age in the reference sample. k, q, and s are the regression intercept, slope, and root mean square error, respectively. SBA is a proportional factor that is equal to the square root of the variance of the actual age explained by the biomarker group in the reference sample. CA is the chronological age. In the BioAge package, the reference sample is NHANES III nonpregnant participants aged 30-75. The algorithm parameter estimates are for males and females. In our study, we used 9 biomarkers, including forced expiratory volume (FEV1), systolic blood pressure, albumin, alkaline phosphatase, blood urea nitrogen, muscle mass, C-reactive protein, glycosylated hemoglobin, and total cholesterol.

The PhenoAge algorithm is derived from multivariate analysis of mortality risk ^[1]^. The initial PhenoAge algorithm was constructed using the mortality elastic net Gompertz regression of 42 biomarkers in NHANES III. Nine biomarkers were selected for this analysis: albumin, alkaline phosphatase, myosin, C-reactive protein, glucose, mean cell volume, red blood cell distribution width, white blood cell count, lymphocyte ratio, and age. The calculation formula is:

$$\mathrm{mortality}\mathrm{risk} ={1-e}^{-e^{xb}\left[ exp\left( {120}_{x\gamma} \right)-1 \right]/\gamma}$$

$$\gamma=0.0076927$$

xb = -19.907 - 0.0336 + albumin + 0.0095 × creatinine + 0.1953 × glucose + 0.0954 × ln (C-reactive protein) - 0.012 × lymphocyte percentage + 0.0268 × mean corpuscular volume + 0.3306 × red cell distribution width + 0.00188 × alkaline phosphatase + 0.0554 × white blood cell count + 0.0804 × chronological age.

$$PhenoAge=141.50225+\frac{\ln\left[ -0.00553\times\ln\left( 1-mortality risk \right) \right]}{0.090165}$$

To quantify the differences between participants in biological aging, we regressed their calculated biological age values to the chronological age at the time of biomarker measurement and calculated the residual value. In the following text, we refer to these residuals as "age acceleration" values to measure biological aging. Computation of biological age values was conducted using the R package ‘BioAge’ (https://github.com/dayoonkwon/BioAge) ^[1]^. Any participants with missing values were excluded from this analysis. Included biomarkers and corresponding UK Biobank data fields are reported in Table 1.

***Assessment of Life’s Essential 8 score***

The LE8 score was calculated to assess cardiovascular health, which included 4 biological (blood lipids, blood glucose, blood pressure, and body mass index) and 4 behavioral (smoking status, physical activity, diet, and sleep) targets.

The measurement of blood lipids and blood glucose was performed at the laboratory center. Blood lipid levels (non-high-density-lipoprotein cholesterol) were calculated as total cholesterol minus HDL cholesterol, which was assessed by enzyme immunoinhibition on a Beckman Coulter AU5800. Blood glucose levels (glycated hemoglobin) were assessed using a plasma sample collected at baseline through a high-performance liquid chromatography method on a Bio-Rad VARIANT II Turbo (Bio-Rad Laboratories, Inc.).

Trained nurses at the assessment center measured systolic and diastolic blood pressure twice, the average levels of which were used in this study. Automated measurements were preferred, and manual measurements were used instead when the former was not available ^[2]^. Body mass index was calculated as weight in kilograms divided by height in meters squared (kg/m2), based on data measured during the assessment visit. The Metabolic Equivalent Task (MET) minutes based on items from the short International Physical Activity Questionnaire (IPAQ) were adopted to assess physical activity. Diet was estimated by calculating the Dietary Approaches to Stop Hypertension diet score ^[3]^ using the data collected from the 24-h dietary recall. Participants with illness, fasting, or other circumstances who reported that their diet was out of the ordinary that day were not included in the 24-h dietary assessments. In this study, the mean values of each dietary component were used. Detailed scoring rules are shown in **eTable 1.** Baseline data on the frequency and duration of nicotine exposure and sleep duration were self-reported and collected through touchscreen questionnaires during the interview process.

Each health metric was given a score between 0 and 100, with a higher score suggesting a higher level of health. Detailed scoring rules are shown in **eeTable 2.** The overall LE8 score was calculated as the unweighted average of the eight scores, ranging from 0 to 100, which was eventually categorized as high (80-100), moderate (50-79), and low (0-49) ^[4]^.

# Reference

[1] Kwon D, Belsky DW. A toolkit for quantification of biological age from blood chemistry and organ function test data: BioAge. Geroscience. 2021;43(6):2795-2808. doi:10.1007/s11357-021-00480-5

[2] Wartolowska  KA﻿, Webb  AJS﻿.  Midlife blood pressure is associated with the severity of white matter hyperintensities: analysis of the UK Biobank cohort study. Eur Heart J. 2021;42(7):750-757. doi:10.1093/eurheartj/ehaa756

[3] Appel  LJ, Moore  TJ, Obarzanek  E, et al; DASH Collaborative Research Group.  A clinical trial of the effects of dietary patterns on blood pressure. N Engl J Med. 1997;336(16):1117-1124. doi:10.1056/NEJM199704173361601

[4] Lloyd-Jones DM, Allen NB, Anderson CAM, et al. Life's Essential 8: Updating and Enhancing the American Heart Association's Construct of Cardiovascular Health: A Presidential Advisory From the American Heart Association. Circulation. 2022;146(5):e18-e43. doi:10.1161/CIR.0000000000001078

# Supplementary Figure and Table Legends

eFigure 1. Study flow chart.

eFigure 1. Correlation heat map of chronological age, biological ages, and age accelerations.

eFigure 2. Relationship between LE8 score and biological aging in difference age.

eTable 1. Definition of the DASH diet score in the UK Biobank.

eTable 2. Methods for evaluating each individual Life's Essential 8 score in the UK Biobank.

eTable 3. Full names and data field IDs of variables for the construction of biological ages.

eTable 4. Subgroup analysis for the association of KDM-BA biological aging with CMD, CMM, dementia, and mortality in individuals with different chronological ages.

eTable 5. Subgroup analysis for the association of PhenoAge biological aging with CMD, CMM, dementia, and mortality in individuals with different chronological ages.

eTable 6. Subgroup analysis for the association of KDM-BA biological aging with CMD, CMM, dementia, and mortality in males.

eTable 7. Subgroup analysis for the association of PhenoAge biological aging with CMD, CMM, dementia, and mortality in females.

eTable 8. Sensitivity analysis for the association of biological aging with CMD, CMM, dementia, and mortality in CMD-free individuals after excluding individuals who had been followed up for less than 2 years.

eTable 9. Sensitivity analysis for the association of biological aging with CMD, CMM, dementia, and mortality in CMD and CVD free individuals.

eTable 10. Subgroup analysis for the association of KDM-BA biological aging with CMM, dementia, and mortality in CMD individuals with different ages.

eTable 11. Subgroup analysis for the association of PhenoAge biological aging with CMM, dementia, and mortality in CMD individuals with different ages.

eTable 12. Subgroup analysis for the association of KDM-BA biological aging with CMM, dementia, and mortality in CMD individuals with different genders.

eTable 13. Subgroup analysis for the association of PhenoAge biological aging with CMM, dementia, and mortality in CMD individuals with different genders.

eTable 14. Sensitivity analysis for the association of PhenoAge biological aging with CMM, dementia, and mortality in CMD individuals after excluding individuals who had been followed up for less than 2 years.

eTable 15. Association of biological aging with CMM, dementia, and mortality in individuals with diabetes, stroke, or ischemic heart disease.

eTable 16. Mediation impact of CMM on relationship of accelerated biological aging with dementia, and mortality.

eTable 17. Mediation impact of CMD on relationship of accelerated biological aging with dementia, and mortality.

eTable 18. Mediation impact of diabetes on relationship of accelerated biological aging with dementia, and mortality.

eTable 19. Mediation impact of ischemic heart disease on relationship of accelerated biological aging with dementia, and mortality.

eTable 20. Mediation impact of stroke on relationship of accelerated biological aging with dementia, and mortality.

eTable 21. Relatinship between LE8 score and biological aging according to multivariate Logistic regression.

eTable 22. Subgroup analysis for relationship between LE8 score and biological aging according to age and gender.

eTable 23. Relatinship between LE8 score and biological aging according to multivariate linear regression.


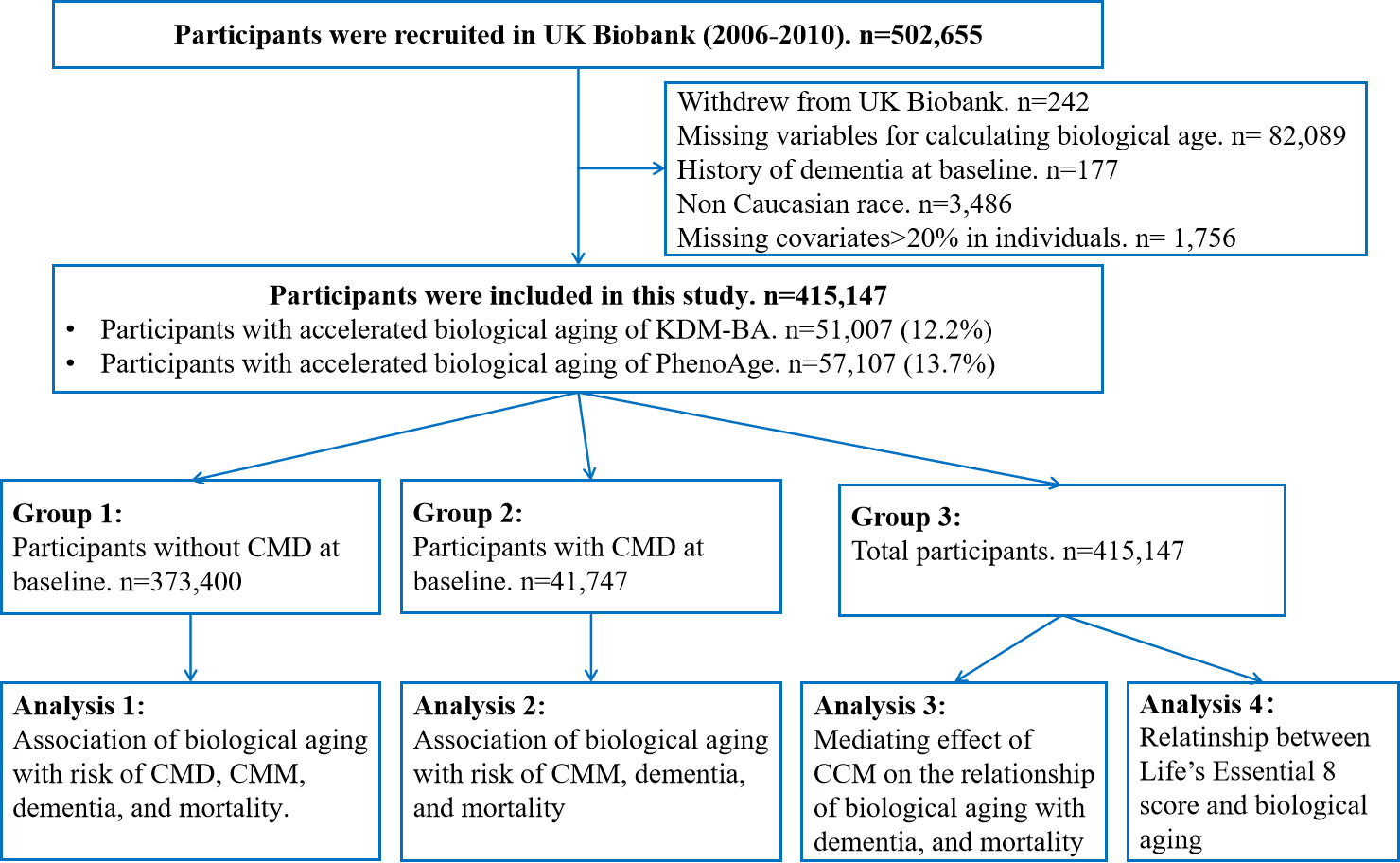


# eFigure 1. Study flow chart. CMD, cardiometabolic disease; CMM, cardiometabolic multimorbidity.


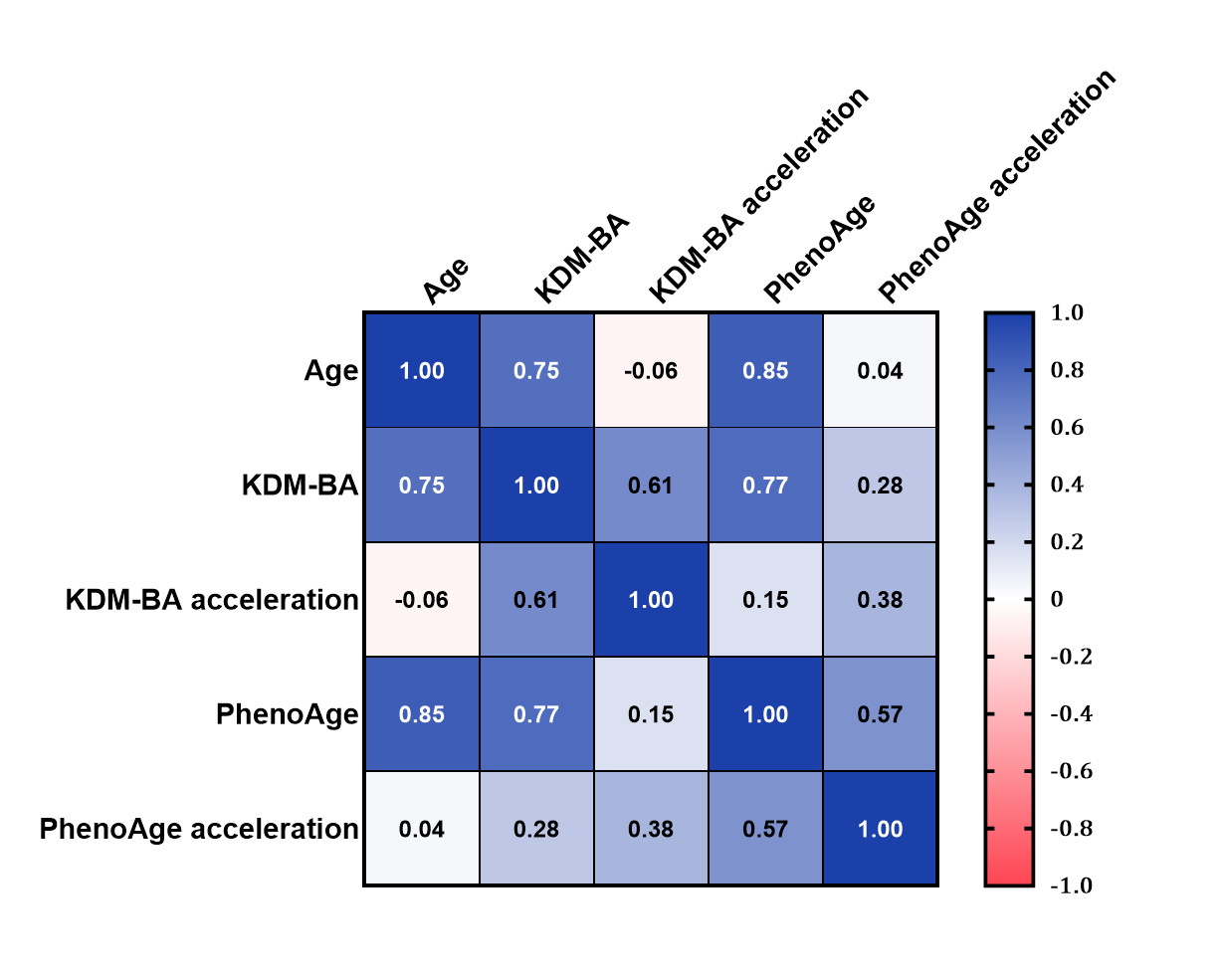


# eFigure 2. Correlation heat map of chronological age, biological ages, and age accelerations.

Pearson correlation coefficients were calculated, and all of the correlations were significant at P<0.001.

**
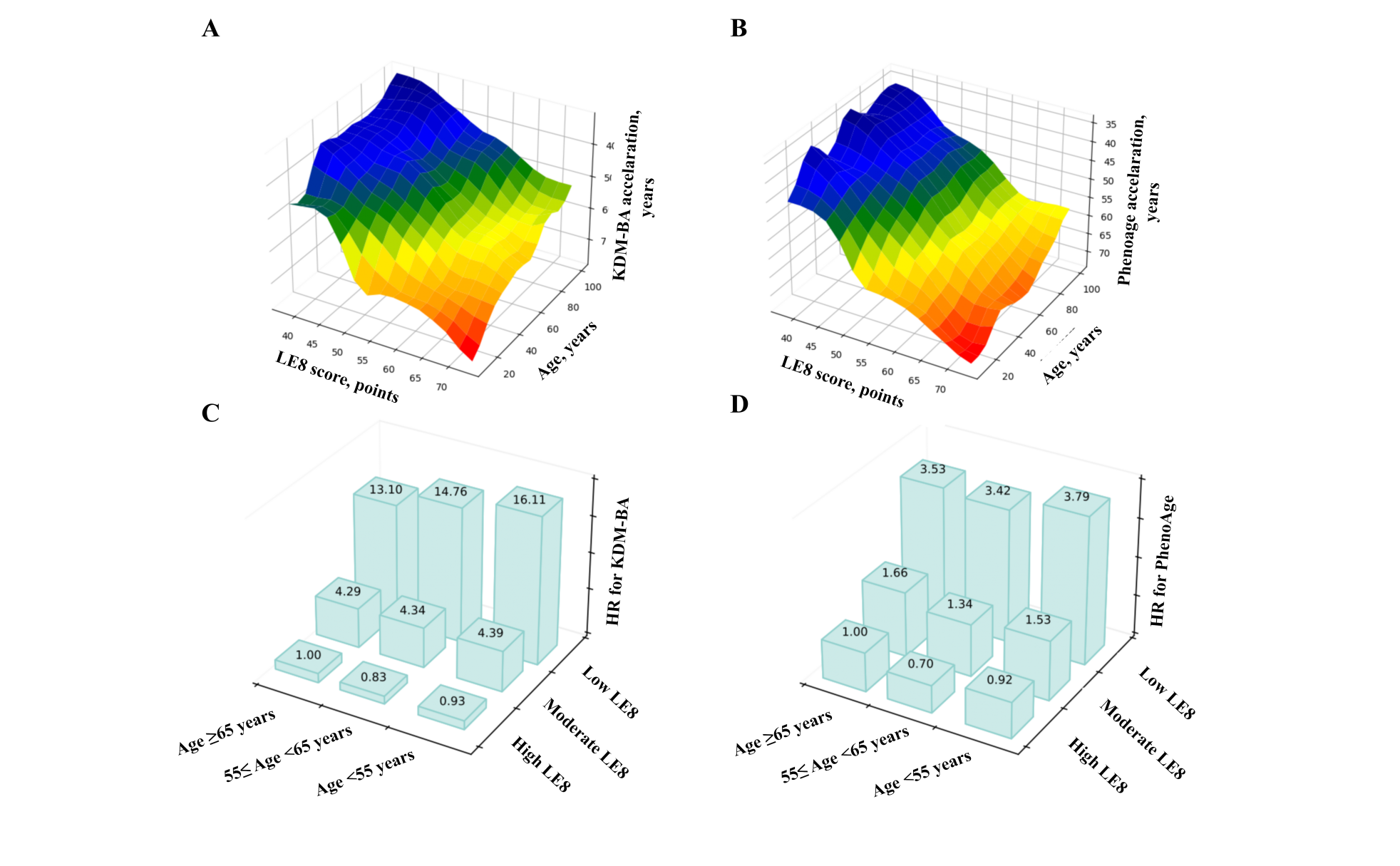
**

# eFigure 3. Relationship between LE8 score and biological aging at different ages.

(A and B) 3D Mesh Plots visualizing differences between accelerated age, chronological age, and LE8 score. (C and D) Adjusted HR of the LE8 score for biological accelerated aging at different ages. LE8, life’s essential 8; HR, hazards ratio.

# eTable 1. Definition of the DASH diet score in the UK Biobank.

| **Diet components** | **Foods** | **Scoring criteria** |
| --- | --- | --- |
| **Healthy components** |  | Q1=1 point  Q2=2 points  Q3=3 points  Q4=4 points  Q5=5 points |
| Fruits | Fresh and dried fruits |  |
| Vegetables | Cooked and raw vegetables |  |
| Whole grains | Bread and cereal |  |
| Nuts and legumes | Nuts and peanut butter, dried beans, peas, tofu |  |
| Low-fat dairy products | Skim milk, yogurt, cottage cheese |  |
| **Unhealthy components** | |  |
| Red and processed meat | Beef, lamb/mutton intake, pork, and processed meat intake | Reverse scoring:  Q1=5 points  Q2=4 points  Q3=3 points  Q4=2 points  Q5=1 point |
| Sugar-sweetened drinks | Carbonated and noncarbonated sweetened beverages |  |
| Estimated 24-hour sodium excretion | Sum of sodium content in foods |  |

The sum of eight diet components is the overall dash diet score, ranging from 8-40 points. Higher scores are related to greater adherence to the DASH diet pattern.

# eTable 2. Methods for evaluating each individual Life's Essential 8 score in the UK Biobank.

| **Life's Essential 8 metric** | **Method of measurement** | **Scoring of Life's Essential 8** | |
| --- | --- | --- | --- |
|  |  | **Points** | **Status** |
| **DASH diet score** | Using dietary data collected from 24-h dietary recalls (1-5 times) conducted using the touch Screen. | 100 | 95th percentile (ideal diet) |
|  |  | 80 | 75th–94th percentile |
|  |  | 50 | 50th–74th percentile |
|  |  | 25 | 25th–49th percentile |
|  |  | 0 | 1st–24th percentile (least ideal quartile) |
| **Physical activity score** | Self-reported weekly minutes of strenuous or moderate physical activity. | 100 | ≥150 minutes |
|  |  | 90 | 120-149 minutes |
|  |  | 80 | 90-119 minutes |
|  |  | 60 | 60-89 minutes |
|  |  | 40 | 30-59 minutes |
|  |  | 20 | 1-29 minutes |
|  |  | 0 | 0 minutes |
| **Tobacco/nicotine exposure score** | Self-reported tobacco use (current smoking status and history of smoking) or secondhand smoke exposure (Participants were asked “Does anyone in your household smoke”. Secondhand smoke exposure was defined as if participants' responses are “yes, one household member smokes” or “Yes, more than one household member smokes”). | 100 | Never smoker |
|  |  | 75 | Former smoker, quit ≥ 5 years |
|  |  | 50 | Former smoker, quit 1–<5 years |
|  |  | 25 | Former smoker, quit <1 years |
|  |  | 0 | Current smoker |
|  |  | If the score is not 0, deduct 20 points for living with active indoor smokers at home.  Furthermore, only individuals who indicated they “smoked on most or all days in the past” have access to information on the precise time to quit smoking. We consider participants who  indicated “smoked occasionally in the past” as equivalent to “Former smoker, quit 1–<5 years”; We consider the participants who indicated “just tried once or twice in the past” as equivalent to “Former smoker, quit ≥ 5 years”. | |
| **Sleep health score** | Self-reported average  hours of sleep per night. | 100 | 7-9 hours |
|  |  | 90 | 9-<10 hours |
|  |  | 70 | 6–<7 hours |
|  |  | 40 | 5–<6 or ≥10 hours |
|  |  | 20 | 4–<5 hours |
|  |  | 0 | < 4 hours |
| **Body mass index score** | Body mass index was calculated as weight (kg) divided by squared height (m^2^)). | 100 | <25 kg/m^2^ |
|  |  | 70 | 25.0-29.9 kg/m^2^ |
|  |  | 30 | 30.0-34.9 kg/m^2^ |
|  |  | 15 | 35.0-39.9 kg/m^2^ |
|  |  | 0 | ≥ 40.0 kg/m^2^ |
| **Blood lipid score** | Non-HDL cholesterol was calculated by total cholesterol minus HDL cholesterol. | 100 | <130 mg/dL |
|  |  | 60 | 130-159 mg/dL |
|  |  | 40 | 160-189 mg/dL |
|  |  | 20 | 190-219 mg/dL |
|  |  | 0 | ≥ 220 mg/dL |
| **Glucose score** | HbA1c was measured by high-performance liquid chromatography (HPLC) analysis on a Bio-Rad VARIANT II Turbo. | 100 | No history of diabetes  HbA1c <5.7 % |
|  |  | 60 | No diabetes and HbA1c 5.7–6.4% |
|  |  | 40 | Diabetes with HbA1c <7.0% |
|  |  | 30 | Diabetes with HbA1c 7.0–7.9% |
|  |  | 20 | Diabetes with HbA1c 8.0–8.9% |
|  |  | 10 | Diabetes with HbA1c 9.0–9.9% |
|  |  | 0 | Diabetes with HbA1c ≥10.0% |
| **Blood pressure score** | The average reading of two BP measurements was used to calculate systolic and diastolic BP. | 100 | <120/<80 mmHg |
|  |  | 75 | 120-129/<80 mmHg |
|  |  | 50 | 130-139 or 80-89 mmHg |
|  |  | 25 | 140-159 or 90-99 mmHg |
|  |  | 0 | ≥ 160 or ≥ 100 mmHg |
|  |  | If the score is not 0, deduct 20 points if participants were in treated level | |

# eTable 3. Full names and data field IDs of variables for the construction of biological ages.

| **Labels in the current study** | **Full name in UK Biobank data dictionary** | **Field ID** |
| --- | --- | --- |
| FEV1 (L) | Forced expiratory volume in 1-second (FEV1) | 3063 |
| Systolic blood pressure (mm Hg) | Systolic blood pressure, automated reading | 4080 |
| White blood cell count (1000 cells/uL) | White blood cell (leukocyte) count | 30000 |
| Red cell distribution width (%) | Red blood cell (erythrocyte) distribution width | 30070 |
| Lymphocyte (%) | Lymphocyte percentage | 30180 |
| Mean cell volume (fL) | Mean sphered cell volume | 30270 |
| Albumin (g/dL) | Albumin | 30600 |
| Alkaline phosphatase (U/L) | Alkaline phosphatase | 30610 |
| Blood urea nitrogen (mg/dL) | Urea | 30670 |
| Total Cholesterol (mg/dL) | Cholesterol | 30690 |
| Creatinine (mg/dL) | Creatinine | 30700 |
| C-reactive protein (mg/dL) | C-reactive protein | 30710 |
| Serum glucose (mg/dL) | Glucose | 30740 |
| Glycated hemoglobin (%) | Glycated hemoglobin (HbA1c) | 30750 |

FEV1, Forced Expiratory Volume in the first second.

# eTable 4. Subgroup analysis for the association of KDM-BA biological aging with CMD, CMM, dementia, and mortality in individuals with different chronological ages.

| **Biological aging** | **Chronological age ≥65** | |  | **Chronological age** <**65** | | **P for interaction** |
| --- | --- | --- | --- | --- | --- | --- |
|  | **Case/number (%)** | **aHR (95%CI)** |  | **Case/number (%)** | **aHR (95%CI)** |  |
| **Diabetes** | 3423/64153 (5.3%) | - |  | 10036/309247 (3.2%) | - | **<0.001** |
| Non-accelerated aging | 2661/57725 (4.6%) | Reference |  | 6748/273294 (2.5%) | Reference |  |
| Accelerated aging | 762/6428 (11.9%) | 1.850 (1.685-2.030) |  | 3288/35953 (9.1%) | 1.986 (1.890-2.086) |  |
| Aging acceleration (per 1 year) | - | 1.048 (1.042-1.053) |  | - | 1.047 (1.044-1.050) |  |
| **Ischemic heart disease** | 7476/64153 (11.7%) | - |  | 16500/309247 (5.3%) | - | **<0.001** |
| Non-accelerated aging | 6438/57725 (11.2%) | Reference |  | 13230/273294 (4.8%) | Reference |  |
| Accelerated aging | 1038/6428 (16.1%) | 1.204 (1.121-1.293) |  | 3270/35953 (9.1%) | 1.339 (1.283-1.398) |  |
| Aging acceleration (per 1 year) | - | 1.017 (1.014-1.021) |  | - | 1.023 (1.020-1.025) |  |
| **Stroke** | 2135/64153 (3.3%) | - |  | 3453/309247 (1.1%) | - | **0.001** |
| Non-accelerated aging | 1836/57725 (3.2%) | Reference |  | 2753/273294 (1.0%) | Reference |  |
| Accelerated aging | 299/6428 (4.7%) | 1.247 (1.092-1.425) |  | 700/35953 (1.9%) | 1.476 (1.344-1.620) |  |
| Aging acceleration (per 1 year) | - | 1.020 (1.012-1.027) |  | - | 1.028 (1.022-1.034) |  |
| **Cardiometabolic disease** | 11629/64153 (18.1%) | - |  | 27331/309247 (8.8%) | - | **<0.001** |
| Non-accelerated aging | 9827/57725 (17%) | Reference |  | 20965/273294 (7.7%) | Reference |  |
| Accelerated aging | 1802/6428 (28%) | 1.386 (1.312-1.465) |  | 6366/35953 (17.7%) | 1.554 (1.505-1.604) |  |
| Aging acceleration (per 1 year) | - | 1.026 (1.023-1.029) |  | - | 1.031 (1.029-1.033) |  |
| **Cardiometabolic multimorbidity** | 1336/64153 (2.1%) | - |  | 2578/309247 (0.8%) | - | **<0.001** |
| Non-accelerated aging | 1057/57725 (1.8%) | Reference |  | 1721/273294 (0.6%) | Reference |  |
| Accelerated aging | 279/6428 (4.3%) | 1.719 (1.482-1.995) |  | 857/35953 (2.4%) | 2.054 (1.867-2.260) |  |
| Aging acceleration (per 1 year) | - | 1.042 (1.033-1.051) |  | - | 1.050 (1.044-1.056) |  |
| **Dementia** | 2140/64153 (3.3%) | - |  | 1582/309247 (0.5%) | - | 0.879 |
| Non-accelerated aging | 1878/57725 (3.3%) | Reference |  | 1346/273294 (0.5%) | Reference |  |
| Accelerated aging | 262/6428 (4.1%) | 1.222 (1.062-1.406) |  | 236/35953 (0.7%) | 1.286 (1.104-1.499) |  |
| Aging acceleration (per 1 year) | - | 1.014 (1.006-1.022) |  | - | 1.019 (1.010-1.028) |  |
| **All-cause mortality** | 7864/64153 (12.3%) | - |  | 12914/309247 (4.2%) | - | 0.814 |
| Non-accelerated aging | 6458/57725 (11.2%) | Reference |  | 10176/273294 (3.7%) | Reference |  |
| Accelerated aging | 1406/6428 (21.9%) | 1.801 (1.690-1.919) |  | 2738/35953 (7.6%) | 1.863 (1.776-1.954) |  |
| Aging acceleration (per 1 year) | - | 1.042 (1.038-1.045) |  | - | 1.046 (1.043-1.049) |  |

Accelerated aging indicates that biological age was greater than chronological age. Aging acceleration indicates biological age minus chronological age.

Models were adjusted by age, sex, education, income, body mass index, physical activity, sleep duration, healthy diet, smoking status, drinking status, diastolic blood pressure, systolic blood pressure, high density lipoprotein cholesterol, low density lipoprotein cholesterol, triglycerides, total cholesterol, and blood glucose. aHR: adjusted hazards ratio; CI: confidence interval; CMD, cardiometabolic disease; CMM, cardiometabolic multimorbidity; aHR, adjusted hazard ratio.

# eTable 5. Subgroup analysis for the association of PhenoAge biological aging with CMD, CMM, dementia, and mortality in individuals with different chronological ages.

| **Biological aging** | **Chronological age ≥65** | |  | **Chronological age** <**65** | | **P for interaction** |
| --- | --- | --- | --- | --- | --- | --- |
|  | **Case/number (%)** | **aHR (95%CI)** |  | **Case/number (%)** | **aHR (95%CI)** |  |
| **Diabetes** | 3423/64153 (5.3%) | - |  | 10036/309247 (3.2%) | - | **<0.001** |
| Non-accelerated aging | 2507/55205 (4.5%) | Reference |  | 7193/274121 (2.6%) | Reference |  |
| Accelerated aging | 916/8948 (10.2%) | 1.533 (1.412-1.665) |  | 2843/35126 (8.1%) | 1.628 (1.551-1.708) |  |
| Aging acceleration (per 1 year) | - | 1.041 (1.035-1.047) |  | - | 1.040 (1.036-1.044) |  |
| **Ischemic heart disease** | 7476/64153 (11.7%) | - |  | 16500/309247 (5.3%) | - | **<0.001** |
| Non-accelerated aging | 6093/55205 (11%) | Reference |  | 13589/274121 (5%) | Reference |  |
| Accelerated aging | 1383/8948 (15.5%) | 1.270 (1.195-1.349) |  | 2911/35126 (8.3%) | 1.381 (1.324-1.440) |  |
| Aging acceleration (per 1 year) | - | 1.024 (1.020-1.028) |  | - | 1.032 (1.029-1.035) |  |
| **Stroke** | 2135/64153 (3.3%) | - |  | 3453/309247 (1.1%) | - | **0.002** |
| Non-accelerated aging | 1736/55205 (3.1%) | Reference |  | 2798/274121 (1.0%) | Reference |  |
| Accelerated aging | 399/8948 (4.5%) | 1.403 (1.253-1.570) |  | 655/35126 (1.9%) | 1.623 (1.485-1.775) |  |
| Aging acceleration (per 1 year) | - | 1.025 (1.017-1.033) |  | - | 1.044 (1.038-1.051) |  |
| **Cardiometabolic disease** | 11629/64153 (18.1%) | - |  | 27331/309247 (8.8%) | - | **<0.001** |
| Non-accelerated aging | 9313/55205 (16.9%) | Reference |  | 21688/274121 (7.9%) | Reference |  |
| Accelerated aging | 2316/8948 (25.9%) | 1.346 (1.284-1.412) |  | 5643/35126 (16.1%) | 1.431 (1.409-1.453) |  |
| Aging acceleration (per 1 year) | - | 1.028 (1.024-1.032) |  | - | 1.037 (1.035-1.039) |  |
| **Cardiometabolic multimorbidity** | 1336/64153 (2.1%) | - |  | 2578/309247 (0.8%) | - | **<0.001** |
| Non-accelerated aging | 979/55205 (1.8%) | Reference |  | 1837/274121 (0.7%) | Reference |  |
| Accelerated aging | 357/8948 (4.0%) | 1.673 (1.471-1.903) |  | 741/35126 (2.1%) | 1.873 (1.716-2.046) |  |
| Aging acceleration (per 1 year) | - | 1.044 (1.035-1.054) |  | - | 1.050 (1.043-1.057) |  |
| **Dementia** | 2140/64153 (3.3%) | - |  | 1582/309247 (0.5%) | - | **0.002** |
| Non-accelerated aging | 1813/55205 (3.3%) | Reference |  | 1322/274121 (0.5%) | Reference |  |
| Accelerated aging | 327/8948 (3.7%) | 1.090 (0.965-1.232) |  | 260/35126 (0.7%) | 1.420 (1.235-1.633) |  |
| Aging acceleration (per 1 year) | - | 1.008 (0.999-1.017) |  | - | 1.025 (1.015-1.035) |  |
| **All-cause mortality** | 7864/64153 (12.3%) | - |  | 12914/309247 (4.2%) | - | **<0.001** |
| Non-accelerated aging | 5854/55205 (10.6%) | Reference |  | 9688/274121 (3.5%) | Reference |  |
| Accelerated aging | 2010/8948 (22.5%) | 1.901 (1.803-2.004) |  | 3226/35126 (9.2%) | 2.196 (2.105-2.291) |  |
| Aging acceleration (per 1 year) | - | 1.057 (1.053-1.060) |  | - | 1.067 (1.065-1.070) |  |

Accelerated aging indicates that biological age was greater than chronological age. Aging acceleration indicates biological age minus chronological age.

Models were adjusted by age, sex, education, income, body mass index, physical activity, sleep duration, healthy diet, smoking status, drinking status, diastolic blood pressure, systolic blood pressure, high density lipoprotein cholesterol, low density lipoprotein cholesterol, triglycerides, total cholesterol, and blood glucose.

aHR: adjusted hazards ratio; CI: confidence interval; CMD, cardiometabolic disease; CMM, cardiometabolic multimorbidity; aHR, adjusted hazard ratio.

# eTable 6. Subgroup analysis for the association of KDM-BA biological aging with CMD, CMM, dementia, and mortality in males.

| **Biological aging** | **Male** | |  | **Female** | | **P for interaction** |
| --- | --- | --- | --- | --- | --- | --- |
|  | **Case/number (%)** | **aHR (95%CI)** |  | **Case/number (%)** | **aHR (95%CI)** |  |
| **Diabetes** | 7283/165037 (4.4%) | - |  | 6176/208363 (3.0%) | - | 0.059 |
| Non-accelerated aging | 4747/140785 (3.4%) | Reference |  | 4662/190234 (2.5%) | Reference |  |
| Accelerated aging | 2536/24252 (10.5%) | 1.883 (1.781-1.991) |  | 1514/18129 (8.4%) | 1.988 (1.851-2.136) |  |
| Aging acceleration (per 1 year) | - | 1.041 (1.037-1.044) |  | - | 1.100 (1.093-1.107) |  |
| **Ischemic heart disease** | 14737/165037 (8.9%) | - |  | 9239/208363 (4.4%) | - | **<0.001** |
| Non-accelerated aging | 11746/140785 (8.3%) | Reference |  | 7922/190234 (4.2%) | Reference |  |
| Accelerated aging | 2991/24252 (12.3%) | 1.289 (1.233-1.347) |  | 1317/18129 (7.3%) | 1.413 (1.321-1.511) |  |
| Aging acceleration (per 1 year) | - | 1.020 (1.018-1.022) |  | - | 1.047 (1.041-1.054) |  |
| **Stroke** | 3068/165037 (1.9%) | - |  | 2520/208363 (1.2%) | - | 0.362 |
| Non-accelerated aging | 2394/140785 (1.7%) | Reference |  | 2195/190234 (1.2%) | Reference |  |
| Accelerated aging | 674/24252 (2.8%) | 1.422 (1.294-1.563) |  | 325/18129 (1.8%) | 1.356 (1.187-1.549) |  |
| Aging acceleration (per 1 year) | - | 1.024 (1.019-1.029) |  | - | 1.036 (1.024-1.048) |  |
| **Cardiometabolic disease** | 22590/165037 (13.7%) | - |  | 16370/208363 (7.9%) | - | **<0.001** |
| Non-accelerated aging | 17219/140785 (12.2%) | Reference |  | 13573/190234 (7.1%) | Reference |  |
| Accelerated aging | 5371/24252 (22.1%) | 1.470 (1.421-1.521) |  | 2797/18129 (15.4%) | 1.631 (1.555-1.712) |  |
| Aging acceleration (per 1 year) | - | 1.027 (1.025-1.029) |  | - | 1.065 (1.061-1.070) |  |
| **Cardiometabolic multimorbidity** | 2403/165037 (1.5%) | - |  | 1511/208363 (0.7%) | - | 0.322 |
| Non-accelerated aging | 1614/140785 (1.1%) | Reference |  | 1164/190234 (0.6%) | Reference |  |
| Accelerated aging | 789/24252 (3.3%) | 1.854 (1.682-2.043) |  | 347/18129 (1.9%) | 2.227 (1.925-2.576) |  |
| Aging acceleration (per 1 year) | - | 1.042 (1.036-1.047) |  | - | 1.104 (1.090-1.118) |  |
| **Dementia** | 1815/165037 (1.1%) | - |  | 1907/208363 (0.9%) | - | 0.401 |
| Non-accelerated aging | 1519/140785 (1.1%) | Reference |  | 1705/190234 (0.9%) | Reference |  |
| Accelerated aging | 296/24252 (1.2%) | 1.207 (1.056-1.381) |  | 202/18129 (1.1%) | 1.304 (1.107-1.536) |  |
| Aging acceleration (per 1 year) | - | 1.012 (1.005-1.018) |  | - | 1.042 (1.028-1.057) |  |
| **All-cause mortality** | 11513/165037 (7.0%) | - |  | 9265/208363 (4.4%) | - | 0.215 |
| Non-accelerated aging | 8638/140785 (6.1%) | Reference |  | 7996/190234 (4.2%) | Reference |  |
| Accelerated aging | 2875/24252 (11.9%) | 1.839 (1.756-1.927) |  | 1269/18129 (7.0%) | 1.768 (1.651-1.892) |  |
| Aging acceleration (per 1 year) | - | 1.042 (1.039-1.044) |  | - | 1.065 (1.059-1.071) |  |

Accelerated aging indicates that biological age was greater than chronological age. Aging acceleration indicates biological age minus chronological age.

Models were adjusted by age, sex, education, income, body mass index, physical activity, sleep duration, healthy diet, smoking status, drinking status, diastolic blood pressure, systolic blood pressure, high density lipoprotein cholesterol, low density lipoprotein cholesterol, triglycerides, total cholesterol, and blood glucose.

aHR: adjusted hazards ratio; CI: confidence interval; CMD, cardiometabolic disease; CMM, cardiometabolic multimorbidity; aHR, adjusted hazard ratio.

# eTable 7. Subgroup analysis for the association of PhenoAge biological aging with CMD, CMM, dementia, and mortality in females.

| **Biological aging** | **Male** | |  | **Female** | | **P for interaction** |
| --- | --- | --- | --- | --- | --- | --- |
|  | **Case/number (%)** | **aHR (95%CI)** |  | **Case/number (%)** | **aHR (95%CI)** |  |
| **Diabetes** | 7283/165037 (4.4%) | - |  | 6176/208363 (3.0%) | - | **<0.001** |
| Non-accelerated aging | 5250/142852 (3.7%) | Reference |  | 4450/186474 (2.4%) | Reference |  |
| Accelerated aging | 2033/22185 (9.2%) | 1.463 (1.383-1.548) |  | 1726/21889 (7.9%) | 1.778 (1.671-1.892) |  |
| Aging acceleration (per 1 year) | - | 1.029 (1.024-1.034) |  | - | 1.049 (1.045-1.054) |  |
| **Ischemic heart disease** | 14737/165037 (8.9%) | - |  | 9239/208363 (4.4%) | - | 0.947 |
| Non-accelerated aging | 11902/142852 (8.3%) | Reference |  | 7780/186474 (4.2%) | Reference |  |
| Accelerated aging | 2835/22185 (12.8%) | 1.321 (1.266-1.379) |  | 1459/21889 (6.7%) | 1.384 (1.305-1.467) |  |
| Aging acceleration (per 1 year) | - | 1.028 (1.025-1.031) |  | - | 1.031 (1.027-1.035) |  |
| **Stroke** | 14737/165037 (8.9%) | - |  | 2520/208363 (1.2%) | - | 0.116 |
| Non-accelerated aging | 2415/142852 (1.7%) | Reference |  | 2119/186474 (1.1%) | Reference |  |
| Accelerated aging | 653/22185 (2.9%) | 1.478 (1.35-1.618) |  | 401/21889 (1.8%) | 1.593 (1.425-1.781) |  |
| Aging acceleration (per 1 year) | - | 1.038 (1.031-1.045) |  | - | 1.036 (1.028-1.043) |  |
| **Cardiometabolic disease** | 22590/165037 (13.7%) | - |  | 16370/208363 (7.9%) | - | **<0.001** |
| Non-accelerated aging | 17793/142852 (12.5%) | Reference |  | 13208/186474 (7.1%) | Reference |  |
| Accelerated aging | 4797/22185 (21.6%) | 1.371 (1.325-1.418) |  | 3162/21889 (14.4%) | 1.559 (1.496-1.626) |  |
| Aging acceleration (per 1 year) | - | 1.031 (1.028-1.033) |  | - | 1.038 (1.035-1.041) |  |
| **Cardiometabolic multimorbidity** | 2403/165037 (1.5%) | - |  | 1511/208363 (0.7%) | - | **0.020** |
| Non-accelerated aging | 1715/142852 (1.2%) | Reference |  | 1101/186474 (0.6%) | Reference |  |
| Accelerated aging | 688/22185 (3.1%) | 1.605 (1.459-1.767) |  | 410/21889 (1.9%) | 2.013 (1.779-2.277) |  |
| Aging acceleration (per 1 year) | - | 1.039 (1.032-1.047) |  | - | 1.061 (1.053-1.069) |  |
| **Dementia** | 1815/165037 (1.1%) | - |  | 1907/208363 (0.9%) | - | 0.312 |
| Non-accelerated aging | 1471/142852 (1.0%) | Reference |  | 1664/186474 (0.9%) | Reference |  |
| Accelerated aging | 344/22185 (1.6%) | 1.160 (1.027-1.312) |  | 243/21889 (1.1%) | 1.288 (1.119-1.481) |  |
| Aging acceleration (per 1 year) | - | 1.008 (0.999-1.018) |  | - | 1.021 (1.012-1.031) |  |
| **All-cause mortality** | 327/8948 (3.7%) | - |  | 9265/208363 (4.4%) | - | 0.698 |
| Non-accelerated aging | 8170/142852 (5.7%) | Reference |  | 7372/186474 (4%) | Reference |  |
| Accelerated aging | 3343/22185 (15.1%) | 1.997 (1.913-2.083) |  | 1893/21889 (8.6%) | 2.092 (1.984-2.206) |  |
| Aging acceleration (per 1 year) | - | 1.063 (1.060-1.065) |  | - | 1.062 (1.059-1.066) |  |

Accelerated aging indicates that biological age was greater than chronological age. Aging acceleration indicates biological age minus chronological age.

Models were adjusted by age, sex, education, income, body mass index, physical activity, sleep duration, healthy diet, smoking status, drinking status, diastolic blood pressure, systolic blood pressure, high density lipoprotein cholesterol, low density lipoprotein cholesterol, triglycerides, total cholesterol, and blood glucose.

aHR: adjusted hazards ratio; CI: confidence interval; CMD, cardiometabolic disease; CMM, cardiometabolic multimorbidity; aHR, adjusted hazard ratio.

# eTable 8. Sensitivity analysis for the association of biological aging with CMD, CMM, dementia, and mortality in CMD-free individuals after excluding individuals who had been followed up for less than 2 years.

| **Biological age** |  | **KDM-BA** |  | |  |  | **PhenoAge** |  |
| --- | --- | --- | --- | --- | --- | --- | --- | --- |
|  | **Case/number (%)** | **aHR (95%CI)** | **P** | |  | **Case/number (%)** | **aHR (95%CI)** | **P** |
| **Diabetes** | 12422/367886 (3.4%) | - | | - |  | 12422/367886 (3.4%) | - | - |
| Non-accelerated aging | 8751/326854 (2.7%) | Reference | | - |  | 9053/325369 (2.8%) | Reference | - |
| Accelerated aging | 3671/41032 (8.9%) | 1.963 (1.877-2.054) | | <0.001 |  | 3369/42517 (7.9%) | 1.593 (1.525-1.663) | <0.001 |
| Aging acceleration (per 1 year) | - | 1.047 (1.044-1.050) | | <0.001 |  | - | 1.039 (1.035-1.042) | <0.001 |
| **Ischemic heart disease** | 20704/367886 (5.6%) | - | | - |  | 20704/367886 (5.6%) | - | - |
| Non-accelerated aging | 17053/326854 (5.2%) | Reference | | - |  | 17124/325369 (5.3%) | Reference | - |
| Accelerated aging | 3651/41032(8.9%) | 1.287 (1.237-1.339) | | <0.001 |  | 3580/42517 (8.4%) | 1.309 (1.260-1.359) | <0.001 |
| Aging acceleration (per 1 year) | - | 1.020 (1.018-1.023) | | <0.001 |  | - | 1.027 (1.024-1.030) | <0.001 |
| **Stroke** | 5011/367886 (1.4%) | - | | - |  | 5011/367886 (1.4%) | - | - |
| Non-accelerated aging | 4151/326854 (1.3%) | Reference | | - |  | 4109/325369 (1.3%) | Reference | - |
| Accelerated aging | 860/41032 (2.1%) | 1.333 (1.229-1.447) | | <0.001 |  | 902/42517 (2.1%) | 1.460 (1.354-1.574) | <0.001 |
| Aging acceleration (per 1 year) | - | 1.022 (1.017-1.027) | | <0.001 |  | - | 1.034 (1.029-1.040) | <0.001 |
| **Single cardiometabolic disease** | 31506/367886 (8.6%) | - | | - |  | 31506/367886 (8.6%) | - | - |
| Non-accelerated aging | 25219/326854(7.7%) | Reference | | - |  | 25423/325369 (7.8%) | Reference | - |
| Accelerated aging | 6287/41032(15.3%) | 1.454 (1.409-1.500) | | <0.001 |  | 6083/42517 (14.3%) | 1.395 (1.354-1.437) | <0.001 |
| Aging acceleration (per 1 year) | - | 1.027 (1.025-1.029) | | <0.001 |  | - | 1.031 (1.029-1.033) | <0.001 |
| **Cardiometabolic multimorbidity** | 3255/367886 (0.9%) | - | | - |  | 3255/367886 (0.9%) | - | - |
| Non-accelerated aging | 2329/326854(0.7%) | Reference | | - |  | 2392/325369 (0.7%) | Reference | - |
| Accelerated aging | 926/41032(2.3%) | 1.940 (1.776-2.118) | | <0.001 |  | 863/42517 (2.0%) | 1.652 (1.519-1.796) | <0.001 |
| Aging acceleration (per 1 year) | - | 1.047 (1.041-1.053) | | <0.001 |  | - | 1.046 (1.039-1.052) | <0.001 |
| **Dementia** | 3541/367886 (1.0%) | - | | - |  | 3541/367886 (1.0%) | - | - |
| Non-accelerated aging | 3081/326854(0.9%) | Reference | | - |  | 3010/325369 (0.9%) | Reference | - |
| Accelerated aging | 460/41032 (1.1%) | 1.220 (1.096-1.358) | | <0.001 |  | 531/42517 (1.2%) | 1.167 (1.060-1.284) | 0.002 |
| Aging acceleration (per 1 year) | - | 1.015 (1.009-1.021) | | <0.001 |  | - | 1.011 (1.004-1.018) | 0.002 |
| **All-cause mortality** | 18774/367886 (5.1%) | - | | - |  | 18774/367886 (5.1%) | - | - |
| Non-accelerated aging | 16634/331019 (5.0%) | Reference | - | |  | 15542/329326 (4.7%) | Reference | - |
| Accelerated aging | 4144/42381 (9.8%) | 1.821 (1.753-1.892) | <0.001 | |  | 5236/44074 (11.9%) | 2.047 (1.980-2.116) | <0.001 |
| Aging acceleration (per 1 year) | - | 1.044 (1.041-1.046) | <0.001 | |  | - | 1.063 (1.061-1.065) | <0.001 |

Accelerated aging indicates that biological age was greater than chronological age. Aging acceleration indicates biological age minus chronological age.

Models were adjusted by age, sex, education, income, body mass index, physical activity, sleep duration, healthy diet, smoking status, drinking status, diastolic blood pressure, systolic blood pressure, high density lipoprotein cholesterol, low density lipoprotein cholesterol, triglycerides, total cholesterol, and blood glucose. aHR: adjusted hazards ratio; CI: confidence interval; CMD, cardiometabolic disease; CMM, cardiometabolic multimorbidity; aHR, adjusted hazard ratio.

# eTable 9. Sensitivity analysis for the association of biological aging with CMD, CMM, dementia, and mortality in CMD and CVD free individuals.

| **Biological age** |  | **KDM-BA** |  | |  |  | **PhenoAge** |  |
| --- | --- | --- | --- | --- | --- | --- | --- | --- |
|  | **Case/number (%)** | **aHR (95%CI)** | **P** | |  | **Case/number (%)** | **aHR (95%CI)** | **P** |
| **Diabetes** | 7331/284109 (2.6%) | - | | - |  | 7331/284109 (2.6%) | - | - |
| Non-accelerated aging | 5257/257411 (2.0%) | Reference | | - |  | 5388/254243 (2.1%) | Reference | - |
| Accelerated aging | 2074/26698 (7.8%) | 2.032 (1.913-2.159) | | <0.001 |  | 1943/29866 (6.5%) | 1.672 (1.578-1.772) | <0.001 |
| Aging acceleration (per 1 year) | - | 1.047 (1.043-1.051) | | <0.001 |  | - | 1.039 (1.034-1.043) | <0.001 |
| **Ischemic heart disease** | 14173/284109 (5.0%) | - | | - |  | 14173/284109 (5.0%) | - | - |
| Non-accelerated aging | 11939/257411 (4.6%) | Reference | | - |  | 11975/254243 (4.7%) | Reference | - |
| Accelerated aging | 2234/26698 (8.4%) | 1.307 (1.243-1.373) | | <0.001 |  | 2198/29866 (7.4%) | 1.280 (1.220-1.342) | <0.001 |
| Aging acceleration (per 1 year) | - | 1.020 (1.017-1.023) | | <0.001 |  | - | 1.026 (1.022-1.029) | <0.001 |
| **Stroke** | 3429/284109 (1.2%) | - | | - |  | 3429/284109 (1.2%) | - | - |
| Non-accelerated aging | 2931/257411 (1.1%) | Reference | | - |  | 2886/254243 (1.1%) | Reference | - |
| Accelerated aging | 498/26698 (1.9%) | 1.249 (1.124-1.387) | | <0.001 |  | 543/29866 (1.8%) | 1.383 (1.257-1.522) | <0.001 |
| Aging acceleration (per 1 year) | - | 1.019 (1.013-1.025) | | <0.001 |  | - | 1.031 (1.024-1.038) | <0.001 |
| **Single cardiometabolic disease** | 21094/284109 (7.4%) | - | | - |  | 21094/284109 (7.4%) | - | - |
| Non-accelerated aging | 17335/257411 (6.7%) | Reference | | - |  | 17356/254243 (6.8%) | Reference | - |
| Accelerated aging | 3759/26698 (14.1%) | 1.455 (1.399-1.514) | | <0.001 |  | 3738/29866 (12.5%) | 1.399 (1.367-1.452) | <0.001 |
| Aging acceleration (per 1 year) | - | 1.026 (1.024-1.028) | | <0.001 |  | - | 1.030 (1.027-1.032) | <0.001 |
| **Cardiometabolic multimorbidity** | 1892/284109 (0.7%) | - | | - |  | 1892/284109 (0.7%) | - | - |
| Non-accelerated aging | 1376/257411 (0.5%) | Reference | | - |  | 1423/254243 (0.6%) | Reference | - |
| Accelerated aging | 516/26698 (1.9%) | 1.997 (1.776-2.246) | | <0.001 |  | 469/29866 (1.6%) | 1.628 (1.453-1.824) | <0.001 |
| Aging acceleration (per 1 year) | - | 1.047 (1.039-1.054) | | <0.001 |  | - | 1.047 (1.039-1.055) | <0.001 |
| **Dementia** | 2356/284109 (0.8%) | - | | - |  | 2356/284109 (0.8%) | - | - |
| Non-accelerated aging | 2076/257411 (0.8%) | Reference | | - |  | 2039/254243 (0.8%) | Reference | - |
| Accelerated aging | 280/26698 (1.0%) | 1.325 (1.156-1.519) | | <0.001 |  | 317/29866 (1.1%) | 1.178 (1.040-1.333) | <0.001 |
| Aging acceleration (per 1 year) | - | 1.014 (1.006-1.021) | | <0.001 |  | - | 1.010 (1.001-1.019) | 0.034 |
| **All-cause mortality** | 13555/284109 (4.8%) | - | | - |  | 13555/284109 (4.8%) | - | - |
| Non-accelerated aging | 11241/257411 (4.4%) | Reference | | - |  | 10476/254243 (4.1%) | Reference | - |
| Accelerated aging | 2314/26698 (8.7%) | 1.822 (1.734-1.916) | | <0.001 |  | 3079/29866 (10.3%) | 2.041 (1.956-2.129) | <0.001 |
| Aging acceleration (per 1 year) | - | 1.043 (1.040-1.046) | | <0.001 |  | - | 1.063 (1.060-1.066) | <0.001 |

Accelerated aging indicates that biological age was greater than chronological age. Aging acceleration indicates biological age minus chronological age.

Models were adjusted by age, sex, education, income, body mass index, physical activity, sleep duration, healthy diet, smoking status, drinking status, diastolic blood pressure, systolic blood pressure, high density lipoprotein cholesterol, low density lipoprotein cholesterol, triglycerides, total cholesterol, and blood glucose.

CVD, cardiovascular disease; aHR: adjusted hazards ratio; CI: confidence interval; CMD, cardiometabolic disease; CMM, cardiometabolic multimorbidity; aHR, adjusted hazard ratio.

# eTable 10. Subgroup analysis for the association of KDM-BA biological aging with CMM, dementia, and mortality in CMD individuals with different ages.

| **Biological aging** | **Chronological age ≥65** | |  | **Chronological age <65** | | **P for interaction** |
| --- | --- | --- | --- | --- | --- | --- |
|  | **Case/number (%)** | **aHR (95%CI)** |  | **Case/number (%)** | **aHR (95%CI)** |  |
| **Participants with single cardiometabolic disease** | | | | | | |
| **Cardiometabolic multimorbidity** | 2274/12382 (18.4%) | - |  | 3412/24208 (14.1%) | - | 0.142 |
| Non-accelerated aging | 1779/10602 (16.8%) | Reference |  | 2248/18889 (11.9%) | Reference |  |
| Accelerated aging | 495/1780 (27.8%) | 1.330 (1.111-1.590) |  | 1164/5319 (21.9%) | 1.622 (1.421-1.851) |  |
| Aging acceleration (per 1 year) | - | 1.027 (1.017-1.038) |  | - | 1.038 (1.030-1.046) |  |
| **Dementia** | 727/12382 (5.9%) | - |  | 403/24208 (1.7%) | - | 0.436 |
| Non-accelerated aging | 611/10602 (5.8%) | Reference |  | 290/18889 (1.5%) | Reference |  |
| Accelerated aging | 116/1780 (6.5%) | 1.266 (1.021-1.569) |  | 113/5319 (2.1%) | 1.677 (1.307-2.152) |  |
| Aging acceleration (per 1 year) | - | 1.014 (1.002-1.025) |  | - | 1.025 (1.015-1.036) |  |
| **All-cause mortality** | 2656/12382 (21.5%) | - |  | 2730/24208 (11.3%) | - | 0.089 |
| Non-accelerated aging | 2003/10602 (18.9%) | Reference |  | 1789/18889 (9.5%) | Reference |  |
| Accelerated aging | 653/1780 (36.7%) | 1.954 (1.772-2.154) |  | 941/5319 (17.7%) | 1.939 (1.770-2.124) |  |
| Aging acceleration (per 1 year) | - | 1.048 (1.042-1.053) |  | - | 1.032 (1.029-1.034) |  |
| **Participants with cardiometabolic multimorbidity** | | | | | | |
| **Dementia** | 192/2161 (8.9%) | - |  | 136/2996 (4.5%) | - | **0.005** |
| Non-accelerated aging | 148/1653 (9.0%) | Reference |  | 72/1977 (3.6%) | Reference |  |
| Accelerated aging | 464/508 (8.7%) | 1.185 (0.815-1.723) |  | 64/1019 (6.3%) | 1.916 (1.287-2.851) |  |
| Aging acceleration (per 1 year) | - | 1.012 (0.992-1.032) |  | - | 1.045 (1.023-1.067) |  |
| **All-cause mortality** | 807/2161 (37.3%) | - |  | 732/2996 (24.4%) | - | 0.955 |
| Non-accelerated aging | 531/1653 (32.1%) | Reference |  | 373/1977 (18.9%) | Reference |  |
| Accelerated aging | 276/508 (54.3%) | 1.954 (1.658-2.302) |  | 359/1019 (35.2%) | 1.940 (1.640-2.295) |  |
| Aging acceleration (per 1 year) | - | 1.042 (1.033-1.051) |  | - | 1.049 (1.040-1.058) |  |

Accelerated aging indicates that biological age was greater than chronological age. Aging acceleration indicates biological age minus chronological age.

Models were adjusted by age, sex, education, income, body mass index, physical activity, sleep duration, healthy diet, smoking status, drinking status, diastolic blood pressure, systolic blood pressure, high density lipoprotein cholesterol, low density lipoprotein cholesterol, triglycerides, total cholesterol, and blood glucose.

aHR: adjusted hazards ratio; CI: confidence interval; CMD, cardiometabolic disease; CMM, cardiometabolic multimorbidity; aHR, adjusted hazard ratio

# eTable 11. Subgroup analysis for the association of PhenoAge biological aging with CMM, dementia, and mortality in CMD individuals with different ages.

| **Biological aging** | **Chronological age ≥65** | |  | **Chronological age <65** | | **P for interaction** |
| --- | --- | --- | --- | --- | --- | --- |
|  | **Case/number (%)** | **aHR (95%CI)** |  | **Case/number (%)** | **aHR (95%CI)** |  |
| **Participants with single cardiometabolic disease** | | | | | | |
| **Cardiometabolic multimorbidity** | 2274/12382 (18.4%) | - |  | 3412/24208 (14.1%) | - | 0.317 |
| Non-accelerated aging | 1427/9012 (15.8%) | Reference |  | 1976/17002 (11.6%) | Reference |  |
| Accelerated aging | 847/3370 (25.1%) | 1.358 (1.165-1.584) |  | 1436/7206 (19.9%) | 1.555 (1.374-1.761) |  |
| Aging acceleration (per 1 year) | - | 1.027 (1.014-1.039) |  | - | 1.045 (1.036-1.055) |  |
| **Dementia** | 727/12382 (5.9%) | - |  | 403/24208 (1.7%) | - | 0.807 |
| Non-accelerated aging | 485/9012 (5.4%) | Reference |  | 252/17002 (1.5%) | Reference |  |
| Accelerated aging | 242/3370 (7.2%) | 1.509 (1.276-1.784) |  | 151/7206 (2.1%) | 1.505 (1.203-1.882) |  |
| Aging acceleration (per 1 year) | - | 1.033 (1.021-1.045) |  | - | 1.037 (1.022-1.053) |  |
| **All-cause mortality** | 2656/12382 (21.5%) | - |  | 2730/24208 (11.3%) | - | 0.649 |
| Non-accelerated aging | 1497/9012 (16.6%) | Reference |  | 1392/17002 (8.2%) | Reference |  |
| Accelerated aging | 1159/3370 (34.4%) | 2.213 (2.037-2.404) |  | 1338/7206 (18.6%) | 2.270 (2.090-2.466) |  |
| Aging acceleration (per 1 year) | - | 1.066 (1.061-1.071) |  | - | 1.066 (1.062-1.070) |  |
| **Participants with cardiometabolic multimorbidity** | | | | | | |
| **Dementia** | 192/2161 (8.9%) | - |  | 136/2996 (4.5%) | - | **0.001** |
| Non-accelerated aging | 107/1143 (9.4%) | Reference |  | 58/1557 (3.7%) | Reference |  |
| Accelerated aging | 85/1018 (8.3%) | 1.011 (0.736-1.388) |  | 78/1439 (5.4%) | 1.518 (1.044-2.208) |  |
| Aging acceleration (per 1 year) | - | 1.022 (0.999-1.045) |  | - | 1.031 (1.012-1.050) |  |
| **All-cause mortality** | 807/2161 (37.3%) | - |  | 732/2996 (24.4%) | - | **0.017** |
| Non-accelerated aging | 325/1143 (28.4%) | Reference |  | 239/1557 (15.4%) | Reference |  |
| Accelerated aging | 482/1018 (47.3%) | 1.796 (1.540-2.096) |  | 493/1439 (34.3%) | 2.241 (1.895-2.650) |  |
| Aging acceleration (per 1 year) | - | 1.059 (1.049-1.069) |  | - | 1.048 (1.041-1.055) |  |

Accelerated aging indicates that biological age was greater than chronological age. Aging acceleration indicates biological age minus chronological age.

Models were adjusted by age, sex, education, income, body mass index, physical activity, sleep duration, healthy diet, smoking status, drinking status, diastolic blood pressure, systolic blood pressure, high density lipoprotein cholesterol, low density lipoprotein cholesterol, triglycerides, total cholesterol, and blood glucose.

aHR: adjusted hazards ratio; CI: confidence interval; CMD, cardiometabolic disease; CMM, cardiometabolic multimorbidity; aHR, adjusted hazard ratio

# eTable 12. Subgroup analysis for the association of KDM-BA biological aging with CMM, dementia, and mortality in CMD individuals with different genders.

| **Biological aging** | **Male** | |  | **Female** | | **P for interaction** |
| --- | --- | --- | --- | --- | --- | --- |
|  | **Case/number (%)** | **aHR (95%CI)** |  | **Case/number (%)** | **aHR (95%CI)** |  |
| **Participants with single cardiometabolic disease** | | | | | | |
| **Cardiometabolic multimorbidity** | 3978/23244 (17.1%) | - |  | 1708/13346 (12.8%) | - | 0.133 |
| Non-accelerated aging | 2712/18124 (15.0%) | Reference |  | 1315/11367 (11.6%) | Reference |  |
| Accelerated aging | 1266/5120 (24.7%) | 1.416 (1.256-1.597) |  | 393/1979 (19.9%) | 1.859 (1.485-2.327) |  |
| Aging acceleration (per 1 year) | - | 1.029 (1.022-1.036) |  | - | 1.091 (1.070-1.112) |  |
| **Dementia** | 722/23244 (3.1%) | - |  | 408/13346 (3.1%) | - | 0.893 |
| Non-accelerated aging | 558/18124 (3.1%) | Reference |  | 343/11367 (3.0%) | Reference |  |
| Accelerated aging | 164/5120 (3.2%) | 1.325 (1.096-1.603) |  | 65/1979 (3.3%) | 1.819 (1.340-2.469) |  |
| Aging acceleration (per 1 year) | - | 1.019 (1.011-1.028) |  | - | 1.020 (1.004-1.036) |  |
| **All-cause mortality** | 3873/23244 (16.7%) | - |  | 1513/13346 (11.3%) | - | **0.023** |
| Non-accelerated aging | 2595/18124 (14.3%) | Reference |  | 1197/11367 (10.5%) | Reference |  |
| Accelerated aging | 1278/5120 (25.0%) | 1.889 (1.753-2.036) |  | 316/1979 (16.0%) | 1.935 (1.704-2.198) |  |
| Aging acceleration (per 1 year) | - | 1.032 (1.029-1.034) |  | - | 1.086 (1.073-1.098) |  |
| **Participants with cardiometabolic multimorbidity** | | | | | | |
| **Dementia** | 235/3793 (6.2%) | - |  | 93/1364 (6.8%) | - | 0.841 |
| Non-accelerated aging | 150/2536 (5.9%) | Reference |  | 70/1094 (6.4%) | Reference |  |
| Accelerated aging | 85/1257 (6.8%) | 1.379 (1.023-1.859) |  | 23/270 (8.5%) | 1.955 (1.108-3.450) |  |
| Aging acceleration (per 1 year) | - | 1.027 (1.001-1.054) |  | - | 1.103 (1.052-1.155) |  |
| **All-cause mortality** | 1222/3793 (32.2%) | - |  | 317/1364 (23.2%) | - | 0.439 |
| Non-accelerated aging | 667/2536 (26.3%) | Reference |  | 237/1094 (21.7%) | Reference |  |
| Accelerated aging | 555/1257 (44.2%) | 2.073 (1.790-2.400) |  | 80/270 (29.6%) | 2.075 (1.520-2.832) |  |
| Aging acceleration (per 1 year) | - | 1.042 (1.036-1.049) |  | - | 1.082 (1.055-1.110) |  |

Accelerated aging indicates that biological age was greater than chronological age. Aging acceleration indicates biological age minus chronological age.

Models were adjusted by age, sex, education, income, body mass index, physical activity, sleep duration, healthy diet, smoking status, drinking status, diastolic blood pressure, systolic blood pressure, high density lipoprotein cholesterol, low density lipoprotein cholesterol, triglycerides, total cholesterol, and blood glucose.

aHR: adjusted hazards ratio; CI: confidence interval; CMD, cardiometabolic disease; CMM, cardiometabolic multimorbidity; aHR, adjusted hazard ratio

# eTable 13. Subgroup analysis for the association of PhenoAge biological aging with CMM, dementia, and mortality in CMD individuals with different genders.

| **Biological aging** | **Male** | |  | **Female** | | **P for interaction** |
| --- | --- | --- | --- | --- | --- | --- |
|  | **Case/number (%)** | **aHR (95%CI)** |  | **Case/number (%)** | **aHR (95%CI)** |  |
| **Participants with single cardiometabolic disease** | | | | | | |
| **Cardiometabolic multimorbidity** | 3978/23244 (17.1%) | - |  | 1708/13346 (12.8%) | - | **<0.001** |
| Non-accelerated aging | 2420/16394 (14.8%) | Reference |  | 983/9620 (10.2%) | Reference |  |
| Accelerated aging | 1558/6850 (22.7%) | 1.283 (1.143-1.441) |  | 725/3726 (19.5%) | 1.984 (1.662-2.368) |  |
| Aging acceleration (per 1 year) | - | 1.028 (1.019-1.038) |  | - | 1.051 (1.04-1.063) |  |
| **Dementia** | 722/23244 (3.1%) | - |  | 408/13346 (3.1%) | - | 0.821 |
| Non-accelerated aging | 468/16394 (2.9%) | Reference |  | 269/9620 (2.8%) | Reference |  |
| Accelerated aging | 254/6850 (3.7%) | 1.453 (1.229-1.719) |  | 139/3726 (3.7%) | 1.622 (1.294-2.033) |  |
| Aging acceleration (per 1 year) | - | 1.036 (1.024-1.048) |  | - | 1.033 (1.018-1.049) |  |
| **All-cause mortality** | 3873/23244 (16.7%) | - |  | 1513/13346 (11.3%) | - | 0.087 |
| Non-accelerated aging | 2042/16394 (12.5%) | Reference |  | 847/9620 (8.8%) | Reference |  |
| Accelerated aging | 1831/6850 (26.7%) | 2.156 (2.012-2.311) |  | 666/3726 (17.9%) | 2.374 (2.124-2.654) |  |
| Aging acceleration (per 1 year) | - | 1.066 (1.062-1.07) |  | - | 1.068 (1.062-1.074) |  |
| **Participants with cardiometabolic multimorbidity** | | | | | | |
| **Dementia** | 235/3793 (6.2%) | - |  | 93/1364 (6.8%) | - | 0.689 |
| Non-accelerated aging | 115/1942 (5.9%) | Reference |  | 50/758 (6.6%) | Reference |  |
| Accelerated aging | 120/1851 (6.5%) | 1.157 (0.871-1.536) |  | 43/606 (7.1%) | 1.246 (0.792-1.96) |  |
| Aging acceleration (per 1 year) | - | 1.022 (1.004-1.04) |  | - | 1.049 (1.019-1.08) |  |
| **All-cause mortality** | 1222/3793 (32.2%) | - |  | 317/1364 (23.2%) | - | 0.257 |
| Non-accelerated aging | 433/1942 (22.3%) | Reference |  | 131/758 (17.3%) | Reference |  |
| Accelerated aging | 789/1851 (42.6%) | 1.935 (1.702-2.199) |  | 186/606 (30.7%) | 2.019 (1.585-2.574) |  |
| Aging acceleration (per 1 year) | - | 1.049 (1.043-1.055) |  | - | 1.064 (1.049-1.079) |  |

Accelerated aging indicates that biological age was greater than chronological age. Aging acceleration indicates biological age minus chronological age.

Models were adjusted by age, sex, education, income, body mass index, physical activity, sleep duration, healthy diet, smoking status, drinking status, diastolic blood pressure, systolic blood pressure, high density lipoprotein cholesterol, low density lipoprotein cholesterol, triglycerides, total cholesterol, and blood glucose.

aHR: adjusted hazards ratio; CI: confidence interval; CMD, cardiometabolic disease; CMM, cardiometabolic multimorbidity; aHR, adjusted hazard ratio

# eTable 14. Sensitivity analysis for the association of PhenoAge biological aging with CMM, dementia, and mortality in CMD individuals after excluding individuals who had been followed up for less than 2 years.

| **Biological age** |  | **KDM-BA** |  |  |  | **PhenoAge** |  |
| --- | --- | --- | --- | --- | --- | --- | --- |
|  | **Case/number (%)** | **aHR (95%CI)** | **P** |  | **Case/number (%)** | **aHR (95%CI)** | **P** |
| **Participants with single cardiometabolic disease** | | | | | | | |
| **Cardiometabolic multimorbidity** | 1500/14283 (10.5%) | - | - |  | 1500/14283 (10.5%) | - | - |
| Non-accelerated aging | 1077/11546 (9.3%) | Reference | - |  | 933/10424 (9.0%) | Reference | - |
| Accelerated aging | 423/2737 (15.5%) | 1.701 (1.511-1.915) | <0.001 |  | 567/3859 (14.7%) | 1.718 (1.542-1.913) | <0.001 |
| Aging acceleration (per 1 year) | - | 1.040 (1.033-1.047) | <0.001 |  | - | 1.050 (1.042-1.058) | <0.001 |
| **Dementia** | 370/14283 (2.6%) | - | - |  | 370/14283 (2.6%) | - | - |
| Non-accelerated aging | 301/11546 (2.6%) | Reference | - |  | 248/10424 (2.4%) | Reference | - |
| Accelerated aging | 69/2737 (2.5%) | 1.249 (0.953-1.637) | 0.106 |  | 122/3859 (3.2%) | 1.531 (1.226-1.911) | <0.001 |
| Aging acceleration (per 1 year) | - | 1.013 (0.998-1.028) | 0.079 |  | - | 1.028 (1.011-1.047) | 0.002 |
| **All-cause mortality** | 1562/14283 (10.9%) | - | - |  | 1562/14283 (10.9%) | - | - |
| Non-accelerated aging | 1137/11546 (9.8%) | Reference | - |  | 912/10424 (8.7%) | Reference | - |
| Accelerated aging | 425/2737 (15.5%) | 1.708 (1.520-1.920) | <0.001 |  | 650/3859 (16.8%) | 2.021 (1.822-2.241) | <0.001 |
| Aging acceleration (per 1 year) | - | 1.038 (1.032-1.045) | <0.001 |  | - | 1.062 (1.054-1.069) | <0.001 |
| **Participants with cardiometabolic multimorbidity** | | | | | | | |
| **Dementia** | 317/5001 (6.3%) | - | - |  | 317/5001 (6.3%) | - | - |
| Non-accelerated aging | 213/3554 (6.0%) | Reference | - |  | 161/2655 (6.1%) | Reference | - |
| Accelerated aging | 104/1447 (7.2%) | 1.580 (1.236-2.019) | <0.001 |  | 156/2346 (6.6%) | 1.235 (0.985-1.547) | 0.067 |
| Aging acceleration (per 1 year) | - | 1.028 (1.015-1.041) | <0.001 |  | - | 1.028 (1.014-1.042) | <0.001 |
| **All-cause mortality** | 1387/5001 (27.7%) | - | - |  | 1387/5001 (27.7%) | - | - |
| Non-accelerated aging | 832/3554 (23.4%) | Reference | - |  | 521/2655 (19.6%) | Reference | - |
| Accelerated aging | 555/1447 (38.4%) | 1.873 (1.673-2.096) | <0.001 |  | 866/2346 (36.9%) | 2.025 (1.812-2.263) | <0.001 |
| Aging acceleration (per 1 year) | - | 1.037 (1.031-1.043) | <0.001 |  | - | 1.049 (1.044-1.055) | <0.001 |

Accelerated aging indicates that biological age was greater than chronological age. Aging acceleration indicates biological age minus chronological age.

Models were adjusted by age, sex, education, income, body mass index, physical activity, sleep duration, healthy diet, smoking status, drinking status, diastolic blood pressure, systolic blood pressure, high density lipoprotein cholesterol, low density lipoprotein cholesterol, triglycerides, total cholesterol, and blood glucose.

aHR: adjusted hazards ratio; CI: confidence interval; CMD, cardiometabolic disease; CMM, cardiometabolic multimorbidity; aHR, adjusted hazard ratio

# eTable 15. Accelerated aging was significantly associated with CMM, dementia, and mortality in individuals with single CMD (diabetes, stroke, or ischemic heart disease).

| **Biological age** |  | **KDM-BA** |  |  |  | **PhenoAge** |  |
| --- | --- | --- | --- | --- | --- | --- | --- |
|  | **Case/number (%)** | **aHR (95%CI)** | **P** |  | **Case/number (%)** | **aHR (95%CI)** | **P** |
| **Diabetes** |  |  |  |  |  |  |  |
| **CMM** | 6817/21341 (31.9%) | - | - |  | 6817/21341 (31.9%) | - | - |
| Non-accelerated aging | 4570/15638 (29.2%) | Reference | - |  | 3500/12870 (27.2%) | Reference | - |
| Accelerated aging | 2247/5703 (39.4%) | 1.489 (1.338-1.656) | <0.001 |  | 3317/8471 (39.2%) | 1.488 (1.348-1.643) | <0.001 |
| Aging acceleration (per 1 year) | - | 1.029 (1.023-1.036) | <0.001 |  | - | 1.046 (1.037-1.054) | <0.001 |
| **Dementia** | 771/21341 (3.6%) |  |  |  | 771/21341 (3.6%) |  |  |
| Non-accelerated aging | 550/15638 (3.5%) | Reference | - |  | 414/12870 (3.2%) | Reference | - |
| Accelerated aging | 221/5703 (3.9%) | 1.477 (1.241-1.757) | <0.001 |  | 357/8471 (4.2%) | 1.407 (1.200-1.649) | <0.001 |
| Aging acceleration (per 1 year) | - | 1.028 (1.017-1.038) | <0.001 |  | - | 1.035 (1.025-1.046) | <0.001 |
| **All-cause mortality** | 3625/21341 (17.0%) |  |  |  | 3625/21341 (17.0%) |  |  |
| Non-accelerated aging | 2210/15638 (14.1%) | Reference | - |  | 1523/12870 (11.8%) | Reference | - |
| Accelerated aging | 1415/5703 (24.8%) | 1.919 (1.779-2.070) | <0.001 |  | 2102/8471 (24.8%) | 2.064 (1.918-2.221) | <0.001 |
| Aging acceleration (per 1 year) | - | 1.049 (1.045-1.053) | <0.001 |  | - | 1.059 (1.056-1.063) | <0.001 |
| **Ischemic heart disease** |  |  |  |  |  |  |  |
| **CMM** | 5796/18510 (31.3%) | - | - |  | 5796/18510 (31.3%) | - | - |
| Non-accelerated aging | 4160/15275 (27.2%) | Reference | - |  | 3255/13326 (24.4%) | Reference | - |
| Accelerated aging | 1636/3235 (50.6%) | 1.771 (1.365-2.298) | <0.001 |  | 2541/5184 (49.0%) | 1.333 (1.047-1.697) | <0.001 |
| Aging acceleration (per 1 year) | - | 1.044 (1.029-1.060) | <0.001 |  | - | 1.029 (1.008-1.050) | 0.005 |
| **Dementia** | 715/18510 (3.9%) |  |  |  | 715/18510 (3.9%) |  |  |
| Non-accelerated aging | 558/15275 (3.7%) | Reference | - |  | 463/13326 (3.5%) | Reference | - |
| Accelerated aging | 157/3235 (4.9%) | 1.644 (1.345-2.009) | <0.001 |  | 252/5184 (4.9%) | 1.425 (1.204-1.687) | <0.001 |
| Aging acceleration (per 1 year) | - | 1.028 (1.017-1.039) | <0.001 |  | - | 1.037 (1.025-1.048) | <0.001 |
| **All-cause mortality** | 3580/18510 (19.3%) |  |  |  | 3580/18510 (19.3%) |  |  |
| Non-accelerated aging | 2518/15275 (16.5%) | Reference | - |  | 1817/13326 (13.6%) | Reference | - |
| Accelerated aging | 1062/3235 (32.8%) | 2.061 (1.898-2.239) | <0.001 |  | 1763/5184 (34.0%) | 2.361 (2.199-2.536) | <0.001 |
| Aging acceleration (per 1 year) | - | 1.051 (1.046-1.055) | <0.001 |  | - | 1.063 (1.059-1.067) | <0.001 |
| **Stroke** |  |  |  |  |  |  |  |
| **CMM** | 1427/7407 (19.3%) | - | - |  | 1427/7407 (19.3%) | - | - |
| Non-accelerated aging | 980/6044 (16.2%) | Reference | - |  | 744/5345 (13.9%) | Reference | - |
| Accelerated aging | 447/1363 (32.8%) | 1.781 (1.327-2.388) | <0.001 |  | - | 1.692 (1.303-2.196) | <0.001 |
| Aging acceleration (per 1 year) | - | 1.045 (1.027-1.064) | <0.001 |  | 683/2062 (33.1%) | 1.036 (1.018-1.055) | <0.001 |
| **Dementia** | 335/7407 (4.5%) |  |  |  | 335/7407 (4.5%) |  |  |
| Non-accelerated aging | 249/6044 (4.1%) | Reference | - |  | 202/5345 (3.8%) | Reference | - |
| Accelerated aging | 86/1363 (6.3%) | 1.680 (1.264-2.231) | <0.001 |  | 133/2062 (6.5%) | 1.640 (1.287-2.088) | <0.001 |
| Aging acceleration (per 1 year) | - | 1.022 (1.012-1.032) | <0.001 |  | - | 1.040 (1.023-1.056) | <0.001 |
| **All-cause mortality** | 1415/7407 (19.1%) |  |  |  | 1415/7407 (19.1%) |  |  |
| Non-accelerated aging | 942/6044 (15.6%) | Reference | - |  | 715/5345 (13.4%) | Reference | - |
| Accelerated aging | 473/1363 (34.7%) | 2.316 (2.038-2.632) | <0.001 |  | 700/2062 (33.9%) | 2.338 (2.086-2.619) | <0.001 |
| Aging acceleration (per 1 year) | - | 1.028 (1.025-1.032) | <0.001 |  | - | 1.069 (1.062-1.075) | <0.001 |

Accelerated aging indicates that biological age was greater than chronological age. Aging acceleration indicates biological age minus chronological age.

Models were adjusted by age, sex, education, income, body mass index, physical activity, sleep duration, healthy diet, smoking status, drinking status, diastolic blood pressure, systolic blood pressure, high density lipoprotein cholesterol, low density lipoprotein cholesterol, triglycerides, total cholesterol, and blood glucose.

CVD, cardiovascular disease; aHR: adjusted hazards ratio; CI: confidence interval; CMD, cardiometabolic disease; CMM, cardiometabolic multimorbidity; aHR, adjusted hazard ratio.

# eTable 16. Significant mediation impact of CMM on relationship of accelerated biological aging with dementia and mortality.

| **Trajectory** | **Total effect** | **Direct effect** |  | **Indirect effect** | | |
| --- | --- | --- | --- | --- | --- | --- |
|  | **HR (95%CI)** | **HR (95%CI)** |  | **HR (95%CI)** | **Mediation (%)** | **P** |
| KDM-BA → CMM → Dementia | 1.364 (1.262-1.474) | 1.307 (1.209-1.413) |  | 1.044 (1.035-1.052) | 13.73% | <0.001 |
| KDM-BA → CMM → Mortality | 2.184 (2.117-2.254) | 2.106 (2.041-2.173) |  | 1.037 (1.033-1.042) | 4.66% | <0.001 |
| PhenoAge → CMM → Dementia | 1.418 (1.323-1.519) | 1.315 (1.226-1.410) |  | 1.078 (1.062-1.094) | 21.56% | <0.001 |
| PhenoAge → CMM → Mortality | 2.738 (2.662-2.817) | 2.598 (2.525-2.674) |  | 1.054 (1.048-1.060) | 5.21% | <0.001 |

A total of 1000 iterations were performed for bootstrapping to estimate 95% bias-corrected confidence interval. The percentage mediated was calculated by log (estimated indirect effect)/ log (estimated total effect).

Models were adjusted by age, sex, education, income, body mass index, physical activity, sleep duration, healthy diet, smoking status, drinking status, diastolic blood pressure, systolic blood pressure, high density lipoprotein cholesterol, low density lipoprotein cholesterol, triglycerides, total cholesterol, and blood glucose.

CI: confidence interval; CMD, cardiometabolic disease; CMM, cardiometabolic multimorbidity; HR, hazards ratio.

# eTable 17. Significant mediation impact of CMD on relationship of accelerated biological aging with dementia and mortality.

| **Trajectory** | **Total effect** | **Direct effect** |  | **Indirect effect** | | |
| --- | --- | --- | --- | --- | --- | --- |
|  | **HR (95%CI)** | **HR (95%CI)** |  | **HR (95%CI)** | **Mediation (%)** | **P** |
| KDM-BA → CMD → Dementia | 1.358 (1.259-1.468) | 1.267 (1.172-1.369) |  | 1.073 (1.064-1.082) | 22.85% | <0.001 |
| KDM-BA → CMD → Mortality | 2.175 (2.108-2.224) | 2.047 (1.984-2.112) |  | 1.063 (1.058-1.068) | 7.83% | <0.001 |
| PhenoAge → CMD → Dementia | 1.394 (1.300-1.495) | 1.229 (1.146-1.319) |  | 1.134 (1.118-1.151) | 37.88% | <0.001 |
| PhenoAge → CMD → Mortality | 2.723 (2.646-2.802) | 2.477 (2.407-2.550) |  | 1.099 (1.092-1.106) | 9.44% | <0.001 |

A total of 1000 iterations were performed for bootstrapping to estimate 95% bias-corrected confidence interval. The percentage mediated was calculated by log (estimated indirect effect)/ log (estimated total effect).

Models were adjusted by age, sex, education, income, body mass index, physical activity, sleep duration, healthy diet, smoking status, drinking status, diastolic blood pressure, systolic blood pressure, high density lipoprotein cholesterol, low density lipoprotein cholesterol, triglycerides, total cholesterol, and blood glucose.

CI: confidence interval; CMD, cardiometabolic disease; CMM, cardiometabolic multimorbidity; HR, hazards ratio.

# eTable 18. Significant mediation impact of diabetes on relationship of accelerated biological aging with dementia and mortality.

| **Trajectory** | **Total effect** | **Direct effect** |  | **Indirect effect** | | |
| --- | --- | --- | --- | --- | --- | --- |
|  | **HR (95%CI)** | **HR (95%CI)** |  | **HR (95%CI)** | **Mediation (%)** | **P** |
| KDM-BA → Diabetes → Dementia | 1.363 (1.261-1.473) | 1.263 (1.168-1.473) |  | 1.080 (1.067-1.1.091) | 24.59% | <0.001 |
| KDM-BA → Diabetes → Mortality | 2.181 (2.113-2.250) | 2.056 (1.992-2.122) |  | 1.061 (1.055-1.066) | 7.55% | <0.001 |
| PhenoAge → Diabetes → Dementia | 1.407 (1.312-1.509) | 1.256 (1.170-1.348) |  | 1.120 (1.100-1.141) | 33.29% | <0.001 |
| PhenoAge → Diabetes → Mortality | 2.740 (2.663-2.819) | 2.547 (2.474-2.622) |  | 1.076 (1.068-1.084) | 7.24% | <0.001 |

A total of 1000 iterations were performed for bootstrapping to estimate 95% bias-corrected confidence interval. The percentage mediated was calculated by log (estimated indirect effect)/ log (estimated total effect).

Models were adjusted by age, sex, education, income, body mass index, physical activity, sleep duration, healthy diet, smoking status, drinking status, diastolic blood pressure, systolic blood pressure, high density lipoprotein cholesterol, low density lipoprotein cholesterol, triglycerides, total cholesterol, and blood glucose.

CI: confidence interval; CMD, cardiometabolic disease; CMM, cardiometabolic multimorbidity; HR, hazards ratio.

# eTable 19. Significant mediation impact of ischemic heart disease on relationship of accelerated biological aging with dementia and mortality.

| **Trajectory** | **Total effect** | **Direct effect** |  | **Indirect effect** | | |
| --- | --- | --- | --- | --- | --- | --- |
|  | **HR (95%CI)** | **HR (95%CI)** |  | **HR (95%CI)** | **Mediation (%)** | **P** |
| KDM-BA → IHD → Dementia | 1.368 (1.266-1.478) | 1.351 (1.250-1.459) |  | 1.013 (1.010-1.016) | 4.08% | <0.001 |
| KDM-BA → IHD → Mortality | 2.185 (2.118-2.254) | 2.156 (2.090-2.224) |  | 1.014 (1.011-1.016) | 17.33% | <0.001 |
| PhenoAge → IHD → Dementia | 1.414 （1.320-1.515） | 1.362 (1.271-1.460) |  | 1.038 (1.031, 1.046) | 10.86% | <0.001 |
| PhenoAge → IHD → Mortality | 2.731 (2.655-2.810) | 2.639 (2.565-2.715) |  | 1.035 (1.031-1.039) | 3.41% | <0.001 |

A total of 1000 iterations were performed for bootstrapping to estimate 95% bias-corrected confidence interval. The percentage mediated was calculated by log (estimated indirect effect)/ log (estimated total effect).

Models were adjusted by age, sex, education, income, body mass index, physical activity, sleep duration, healthy diet, smoking status, drinking status, diastolic blood pressure, systolic blood pressure, high density lipoprotein cholesterol, low density lipoprotein cholesterol, triglycerides, total cholesterol, and blood glucose.

CI: confidence interval; CMD, cardiometabolic disease; CMM, cardiometabolic multimorbidity; IHD, ischemic heart disease, HR, hazards ratio.

# eTable 20. Significant mediation impact of stroke on relationship of accelerated biological aging with dementia and mortality.

| **Trajectory** | **Total effect** | **Direct effect** |  | **Indirect effect** | | |
| --- | --- | --- | --- | --- | --- | --- |
|  | **HR (95%CI)** | **HR (95%CI)** |  | **HR (95%CI)** | **Mediation (%)** | **P** |
| KDM-BA → stroke → Dementia | 1.367 (1.266-1.477) | 1.349 (1.249-1.457) |  | 1.014 (1.010-1.017) | 4.31% | <0.001 |
| KDM-BA → stroke → Mortality | 2.185 (2.119-2.255) | 2.165 (2.098-2.233) |  | 1.010 (1.008-1.012) | 1.25% | <0.001 |
| PhenoAge → stroke → Dementia | 1.413 (1.319-1.514) | 1.377 (1.285-1.475) |  | 1.027 (1.020-1.033) | 7.60% | <0.001 |
| PhenoAge → stroke → Mortality | 2.741 (2.664-2.820) | 2.694 (2.619-2.771) |  | 1.018 (1.015-1.020) | 1.72% | <0.001 |

A total of 1000 iterations were performed for bootstrapping to estimate 95% bias-corrected confidence interval. The percentage mediated was calculated by log (estimated indirect effect)/ log (estimated total effect).

Models were adjusted by age, sex, education, income, body mass index, physical activity, sleep duration, healthy diet, smoking status, drinking status, diastolic blood pressure, systolic blood pressure, high density lipoprotein cholesterol, low density lipoprotein cholesterol, triglycerides, total cholesterol, and blood glucose.

CI: confidence interval; CMD, cardiometabolic disease; CMM, cardiometabolic multimorbidity; IHD, ischemic heart disease, HR, hazards ratio.

# eTable 21. Negative relatinship between LE8 score and biological aging according to multivariate Logistic regression.

| **Life's Essential 8** | **KDM-BA** |  |  | **PhenoAge** |  |
| --- | --- | --- | --- | --- | --- |
|  | **aOR (95%CI)** | **P** |  | **aOR (95%CI)** | **P** |
| **Life essential 8 scroe** |  |  |  |  |  |
| High (80–100) | reference | - |  | reference | - |
| Moderate (50–79) | 4.835 (4.546-5.143) | <0.001 |  | 1.729 (1.665-1.794) | <0.001 |
| Low (0–49) | 16.615 (15.589-17.709) | <0.001 |  | 4.190 (4.021-4.365) | <0.001 |
| **Life's Essential 8 components*** |  |  |  |  |  |
| Blood pressure | 4.528 (4.324-4.741) | <0.001 |  | 1.042 (1.014-1.071) | 0.004 |
| Blood glucose | 1.742 (1.702-1.782) | <0.001 |  | 2.461 (2.411-2.513) | <0.001 |
| Blood lipids | 1.417 (1.381-1.454) | <0.001 |  | 0.667 (0.653-0.681) | <0.001 |
| Physical activity | 1.224 (1.2-1.248) | <0.001 |  | 1.28 (1.257-1.304) | <0.001 |
| Sleep health | 1.238 (1.213-1.263) | <0.001 |  | 1.248 (1.224-1.272) | <0.001 |
| BMI | 2.001 (1.951-2.052) | <0.001 |  | 1.62 (1.585-1.657) | <0.001 |
| Nicotine exposure | 1.705 (1.659-1.752) | <0.001 |  | 2.468 (2.408-2.53) | <0.001 |
| Diet | 1.412 (1.368-1.457) | <0.001 |  | 1.435 (1.394-1.478) | <0.001 |

*Each individual Life's Essential 8 components with 100 points were used as a reference group. The detailed scoring rules were presented at **eTable 2**.

All models were adjusted for sex, age, income, education, and 8 factors in LE8. aOR, adjusted odds ratio.

# eTable 22. Subgroup analysis for relationship between LE8 score and biological aging according to age and gender.

| **LE 8 score** | **KDM-BA accelerated aging** | |  | **PhenoAge accelerated aging** | |
| --- | --- | --- | --- | --- | --- |
|  | **aOR (95%CI)** | **P for interaction** |  | **aOR (95%CI)** | **P for interaction** |
| **Chronological age ≥65 years** |  | <0.001 |  |  | <0.001 |
| High LE 8 score (80–100) | reference |  |  | reference |  |
| Moderate LE 8 score (50–79) | 4.286 (3.611-5.087) |  |  | 1.662 (1.521-1.816) |  |
| Low LE 8 score (0–49) | 13.144 (11.029-15.666) |  |  | 3.534 (3.209-3.892) |  |
| **55≤ Chronological age <65 years** |  |  |  |  |  |
| High LE 8 score(80–100) | reference |  |  | reference |  |
| Moderate LE 8 score (50–79) | 5.237 (4.673-5.869) |  |  | 1.859 (1.738-1.988) |  |
| Low LE 8 score (0–49) | 17.813 (15.858-20.008) |  |  | 4.672 (4.348-5.019) |  |
| **Chronological age <55 years** |  |  |  |  |  |
| High LE 8 score (80–100) | reference |  |  | reference |  |
| Moderate LE 8 score (50–79) | 4.780 (4.406-5.185) |  |  | 1.840 (1.745-1.939) |  |
| Low LE 8 score (0–49) | 17.528 (16.086-19.100) |  |  | 4.879 (4.591-5.185) |  |
| **Male** |  | <0.001 |  |  | <0.001 |
| High LE 8 score (80–100) | reference |  |  | reference |  |
| Moderate LE 8 score (50–79) | 3.442 (3.172-3.734) |  |  | 1.752 (1.648-1.862) |  |
| Low LE 8 score (0–49) | 11.048 (10.16-12.014) |  |  | 4.136 (3.876-4.412) |  |
| **Female** |  |  |  |  |  |
| High LE 8 score (80–100) | reference |  |  | reference |  |
| Moderate LE 8 score (50–79) | 6.947 (6.318-7.638) |  |  | 1.864 (1.778-1.955) |  |
| Low LE 8 score (0–49) | 26.849 (24.328-29.632) |  |  | 4.88 (4.618-5.156) |  |

LE8, life essential 8; aOR, adjusted odds ratio.

# eTable 23. Significant relatinship between LE8 score and biological aging according to multivariate linear regression.

| **Life's Essential 8** | **KDM-BA** | |  | **PhenoAge** | |
| --- | --- | --- | --- | --- | --- |
|  | **β (95%CI)** | **P** |  | **β (95%CI)** | **P** |
| **Life's Essential 8 score** | -0.201 (-0.202,-0.199) | <0.001 |  | -0.089 (-0.090, -0.087) | <0.001 |
| **Life's Essential 8 components** |  |  |  |  |  |
| Blood pressure, mmhg | 0.150 (0.149, 0.151) | <0.001 |  | -0.001 (0.002, 0.000) | <0.001 |
| Blood glucose, mmol/l | 0.039 (0.038, 0.039) | <0.001 |  | 0.060 (0.060, 0.061) | <0.001 |
| Blood lipids, mmol/l | 0.022 (0.022, 0.022) | <0.001 |  | 0.009 (0.009,0.010) | <0.001 |
| Physical activity, MET/week | -0.002 (-0.002, -0.002) | <0.001 |  | -0.002 (-0.002, -0.002) | <0.001 |
| Sleep duration, hours | 0.010 (-0.006，0.025) | 0.222 |  | 0.077 (0.063, 0.090) | <0.001 |
| Body mass index, kg/m^2^ | 0.210 (0.206，0.214) | <0.001 |  | 0.201 (0.197, 0.204) | <0.001 |
| Nicotine exposure, years | 0.711 (0.686, 0.736) | <0.001 |  | 0.777 (0.755, 0.799) | <0.001 |
| Dash diet score, points | -0.387 (-0.399，-0.374) | <0.001 |  | -0.397 (-0.408,-0.386) | <0.001 |

All models were adjusted for sex, age, income, education, and 8 factors in LE8. aOR, adjusted odds ratio.
